# Supplementary material for: Type I interferon exacerbates Mycobacterium tuberculosis induced human macrophage death
Source: EMBO Rep. 2024 Jun 12;25(7):15. doi: 10.1038/s44319-024-00171-0 (PMC11239827; doi:10.1038/s44319-024-00171-0)
Supplement: Supplementary file 1 — Appendix [file 44319_2024_171_MOESM1_ESM.pdf]

Appendix

Table of Contents

Appendix Figure S1 ....page 2
Appendix Figure S2 ....page 4
Appendix Figure S3 ....page 5
Appendix Figure S4 ....page 6

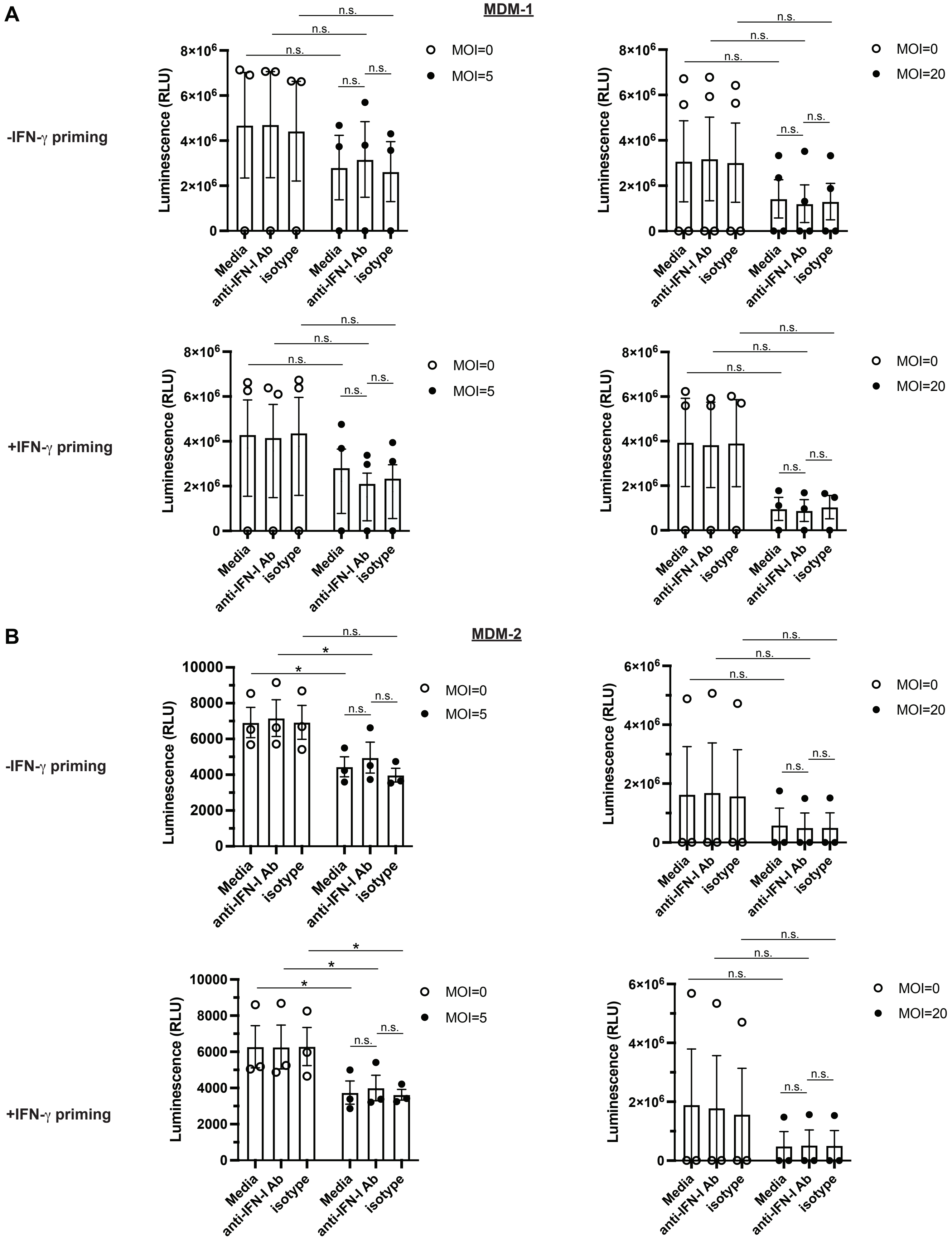
A
MDM-1
-IFN-γ priming
+IFN-γ priming
B
MDM-2
Luminescence (RLU)
MOI=0
MOI=5
MOI=20
Media
anti-IFN-I Ab
isotype
n.s.
*

Appendix Fig. S1 Anti-IFN-I neutralizing antibodies do not inhibit Mtb-induced death of MDMs. MDM-1 (**A**) or MDM-2 (**B**) primed with (bottom) or without (top) 2.5 ng/mL IFN-γ, treated with 1:1000 dilution of neutralizing antibodies (Ab) against IFN-I and IFNAR, isotype control Ab (IgG2a) or medium alone (vehicle control) for 2 hours pre-infection, and infected with Mtb strain H37Rv. 4 hours post infection, extracellular Mtb was washed out and medium was replaced, along with the respective Abs. ATP was measured to determine cell viability at 4 days post infection (left) or 2 days post infection (right). Data Information: Bar graphs (**A** and **B**) report the mean ± SEM of the ATP levels. Each ● or ○ represents the average value of 3 technical replicates for a different donor, n = 3 (except (**A**) top right: n = 4), while n indicates biological replicates. Statistical significance was determined using a paired two-tailed, *t*-test (*$p < 0.5$; n.s. indicates no statistical significance).

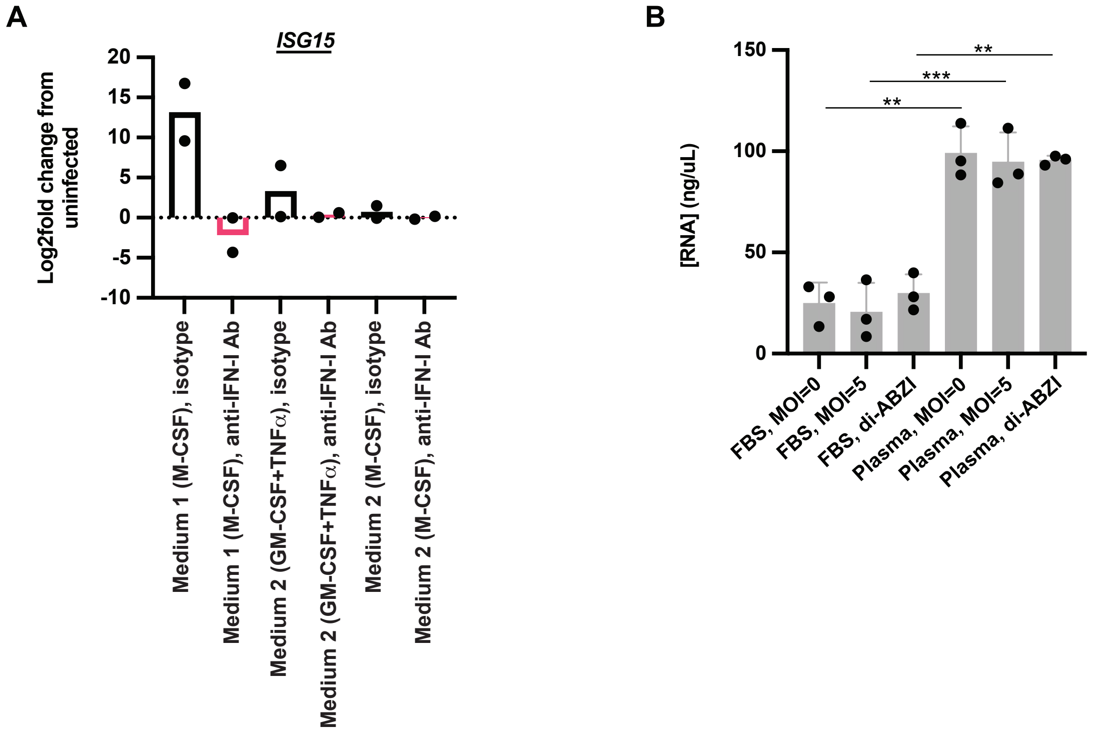

Appendix Fig. S2 Type of medium determines the level of ISG upregulation upon Mtb infection of MDMs and the basal expression of RNA in MDM. (**A**) MDMs were cultured in medium 1 or medium 2 for 1 week under 10% oxygen. MDMs that were differentiated with 10 ng/mL M-CSF instead of GM-CSF were treated with 1:1000 dilution anti-IFN-I neutralizing Abs or IgG2a isotype Ab for 2 h pre-infection, infected with Mtb H37Rv at MOI of 5 and again treated with the respective Ab after Mtb was washed out 4 h post infection. Lysates were collected 1 d post infection. ISG15 expression was measured by RT-qPCR. Bar graphs report the mean ± standard error of mean (SEM). Each ● represents the average value of 4 technical replicates per donor. (**B**) MDMs (n = 3) were differentiated under method 1, except that they were cultured with 10% FBS or 40% autologous human plasma and infected with Mtb H37Rv. Lysates were collected 1 d post infection. RNA concentration was measured by nanodrop or Qubit. Data Information: Bar graphs report the mean ± standard error of mean (SEM). Each ● represents the average value of one technical replicate for one donor. Statistical significance was determined using a two-tailed paired, *t*-test (**$p < 0.01$; ***$p < 0.001$). n indicates biological replicates.

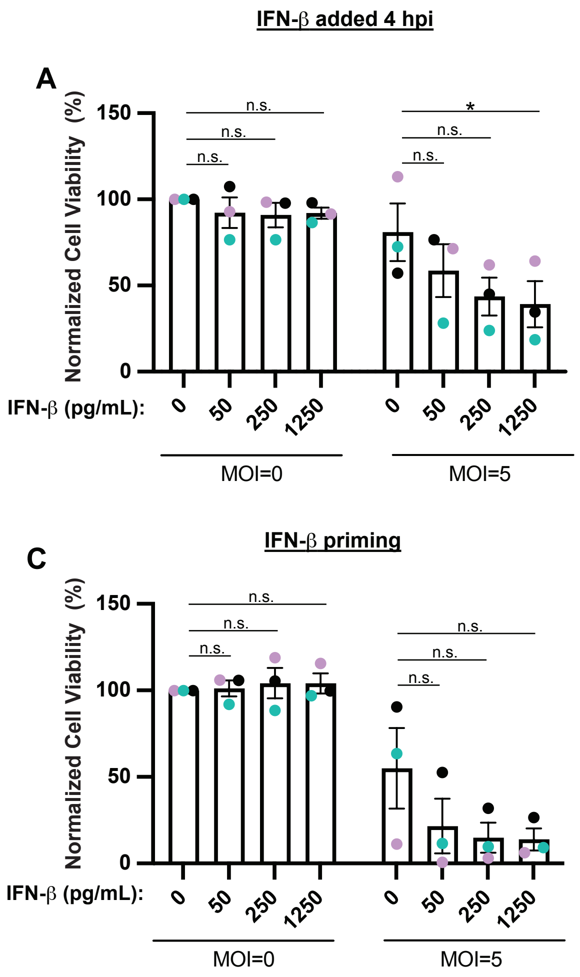

Appendix Fig. S3 Exogenous IFN-I exacerbates Mtb-induced death of MDM-1.
(**A**) IFN-β or (**B**) IFN-α were added to Mtb H37Rv infected MDM-1 (n=3) at 4 h post infection after extracellular Mtb was washed out. Cell viability was determined by CellTiter Glo assay at 2 d post infection. MDM-1 were primed with (**C**) IFN-β or (**D**) IFN-α; 1 day later MDM-1 (n=3) were infected with Mtb H37Rv. Cell viability was measured by CellTiter Glo assay at 2 d post infection. Data Information: (**A**-**D**) Cell viability was normalized to values for the respective uninfected, unstimulated or non-primed MDMs. Bar graphs represent mean ± SEM of the normalized cell viability. Each colored ● represents a different donor, and each ● represents the average value of 3 technical replicates per donor. Cells from each donor were tested in independent experiments, n indicates biological replicates. Statistical significance was determined using a paired, two-tailed *t*-test (*$p < 0.5$; **$p < 0.01$; n.s. indicates no statistical significance).

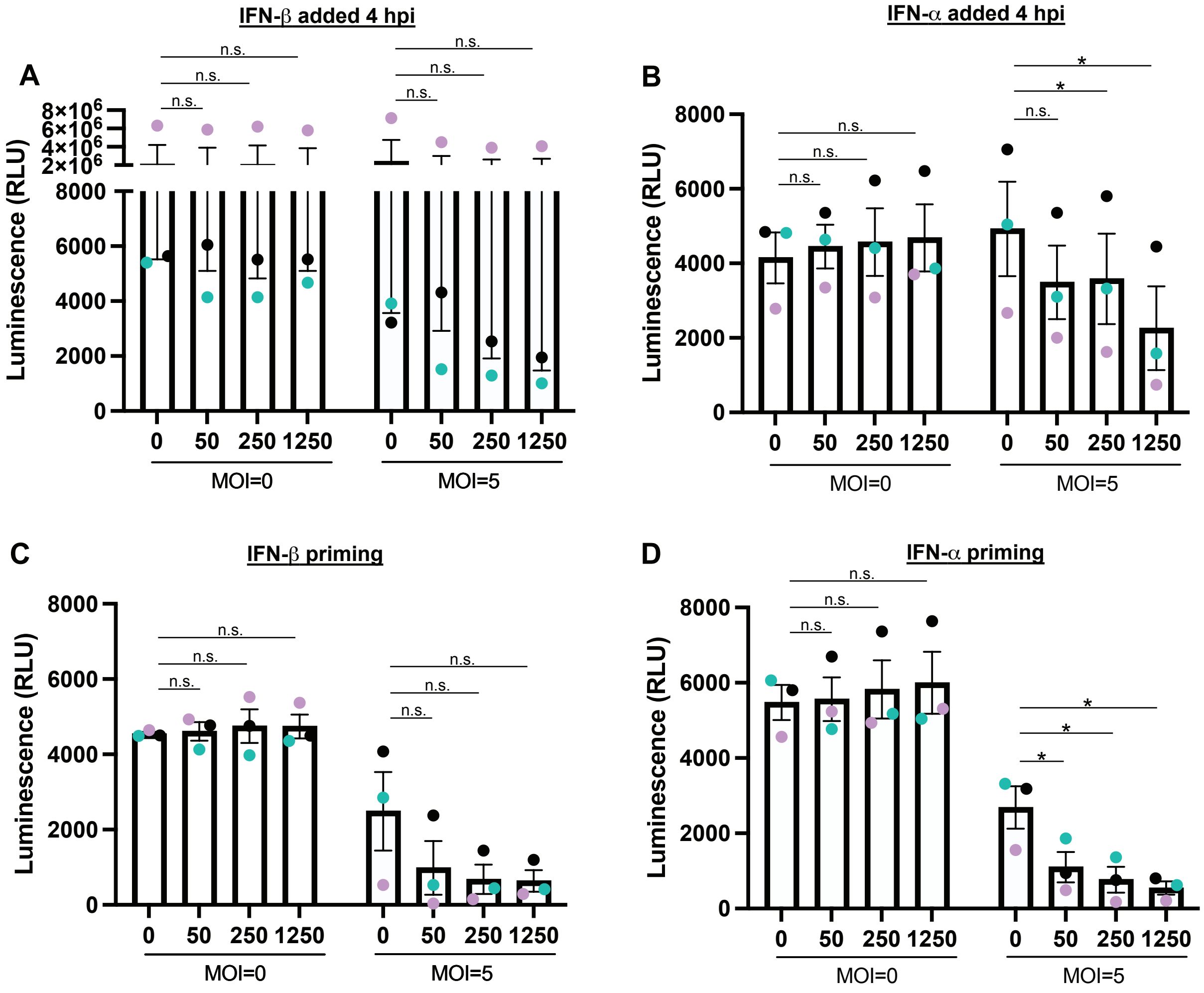

Appendix Fig. S4 Exogeneous IFN-I exacerbates Mtb-Induced death of MDM-1.
(**A**) IFN-β or (**B**) IFN-α were added to Mtb H37Rv infected MDM-1 at 4 h post infection after extracellular Mtb was washed out. ATP was measured to determine cell viability at 2 d post infection. MDM-1 were primed with (**C**) IFN-β or (**D**) IFN-α; 1 day later MDM-1 were infected with Mtb H37Rv. ATP was measured to determine cell viability at 2 d post infection. Data Information: (**A-D**) Bar graphs represent mean ± SEM of ATP levels. Each colored ● represents a different donor, and each ● represents the average value of 3 technical replicates per donor. Cells from each donor were tested in independent experiments, n indicates biological replicates. Statistical significance was determined using a paired, two-tailed *t*-test (*$p < 0.5$; **$p < 0.01$).
