# Supplementary figures and images for: Type I interferon exacerbates Mycobacterium tuberculosis induced human macrophage death
Source: EMBO Rep. 2024 Jun 12;25(7):15. doi: 10.1038/s44319-024-00171-0 (PMC11239827; doi:10.1038/s44319-024-00171-0)

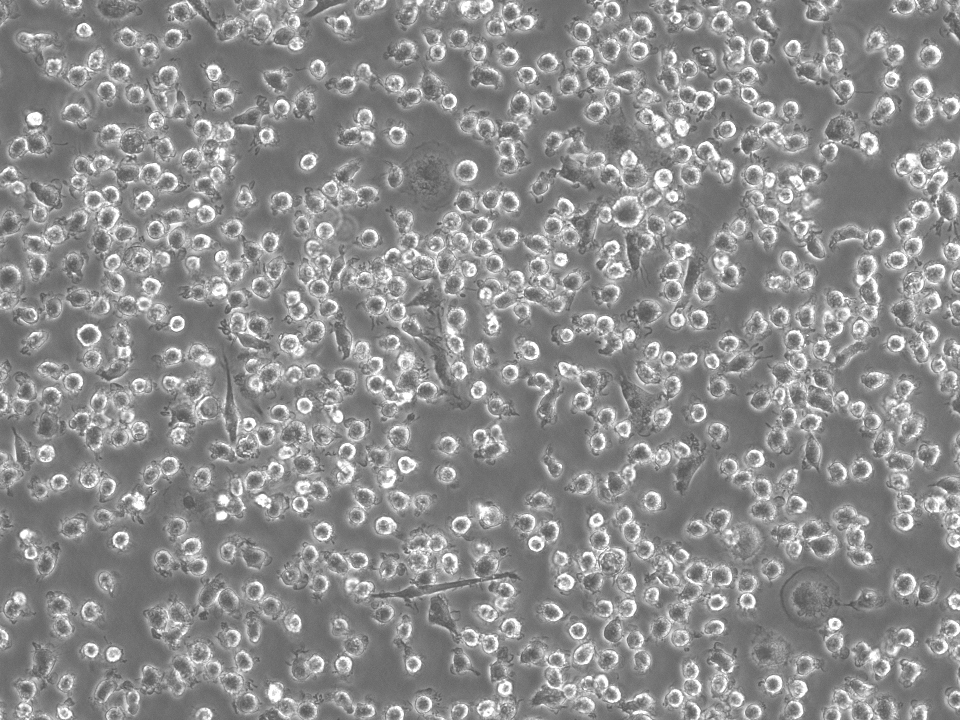

Supplement: Supplementary file 6 — Source data Fig. 4 [file 44319_2024_171_MOESM6_ESM.zip › Figure 4C/Fig. 4C_ bottom, left.tif]

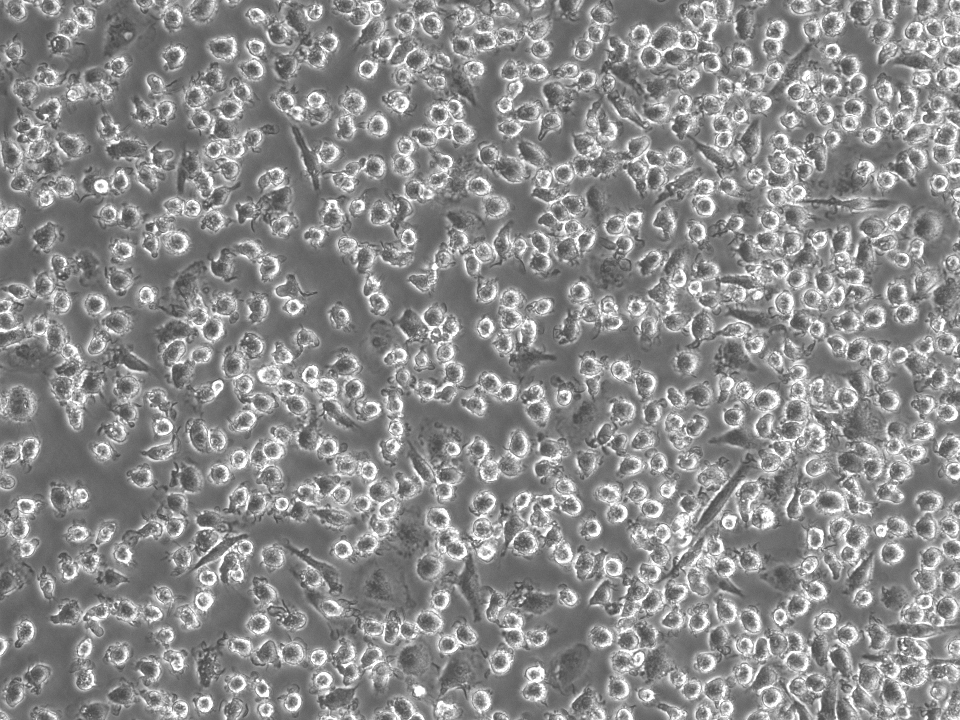

Supplement: Supplementary file 6 — Source data Fig. 4 [file 44319_2024_171_MOESM6_ESM.zip › Figure 4C/Fig. 4C_bottom, left, replicates/Fig. 4C_bottom, left, replicate 2.tif]

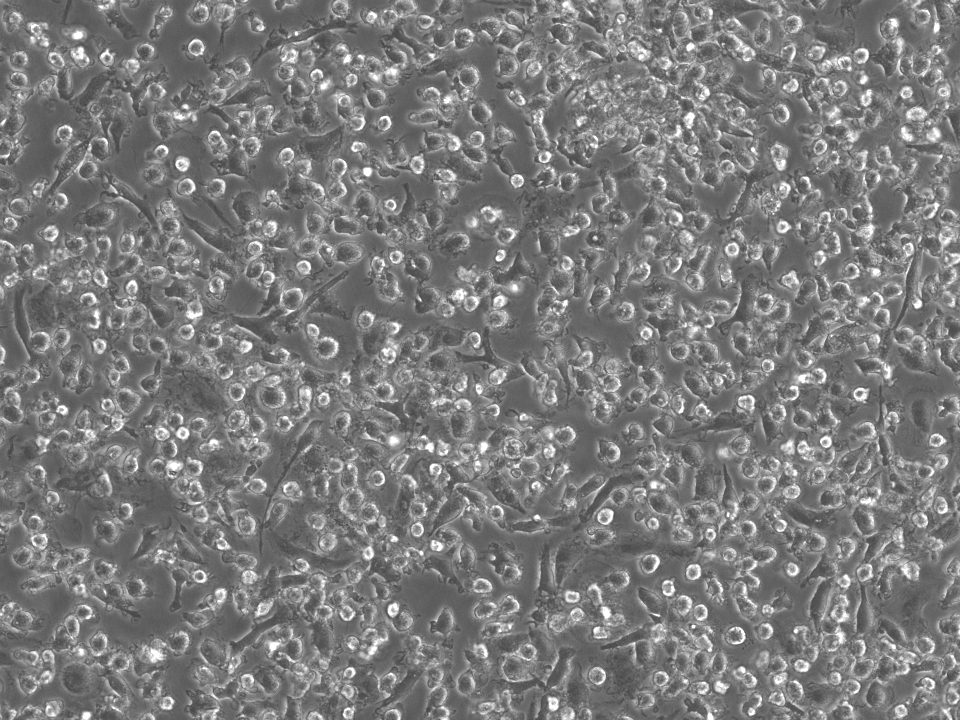

Supplement: Supplementary file 6 — Source data Fig. 4 [file 44319_2024_171_MOESM6_ESM.zip › Figure 4C/Fig. 4C_bottom, left, replicates/Fig. 4C_bottom, left, replicate 3.tif]

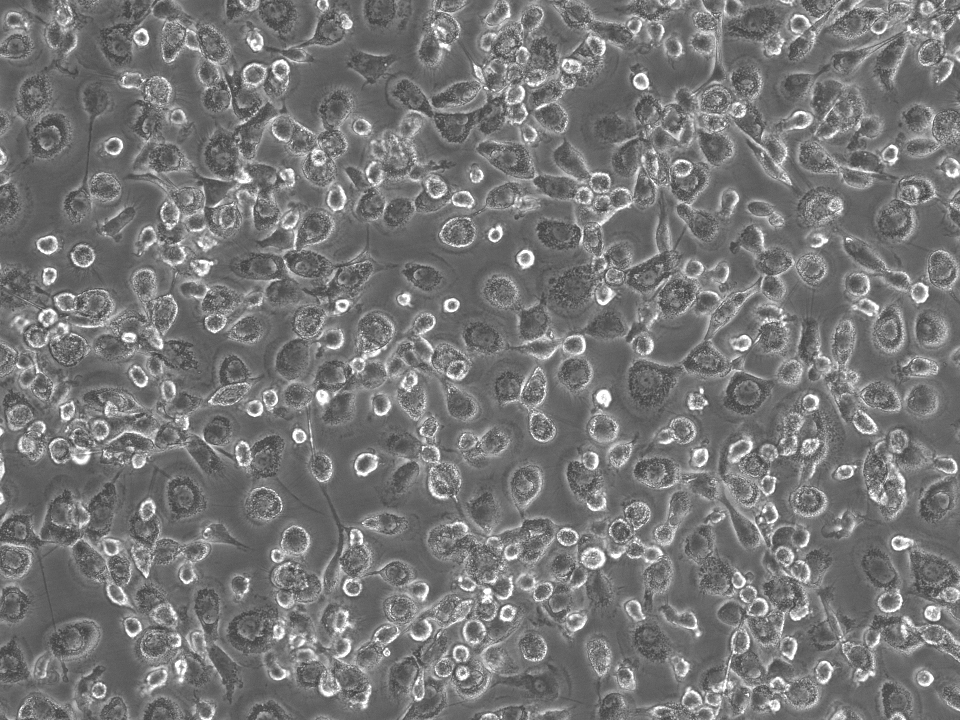

Supplement: Supplementary file 6 — Source data Fig. 4 [file 44319_2024_171_MOESM6_ESM.zip › Figure 4C/Fig. 4C_bottom, right, replicates/Fig. 4C_bottom, right, replicate 2.tif]

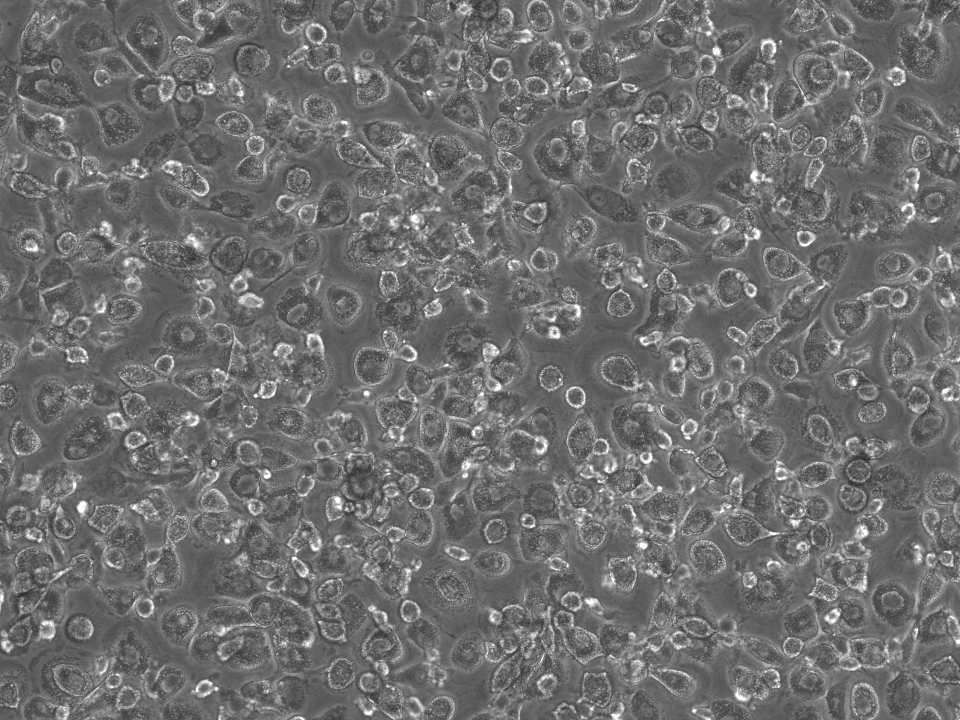

Supplement: Supplementary file 6 — Source data Fig. 4 [file 44319_2024_171_MOESM6_ESM.zip › Figure 4C/Fig. 4C_bottom, right, replicates/Fig. 4C_bottom, right, replicate 3.tif]

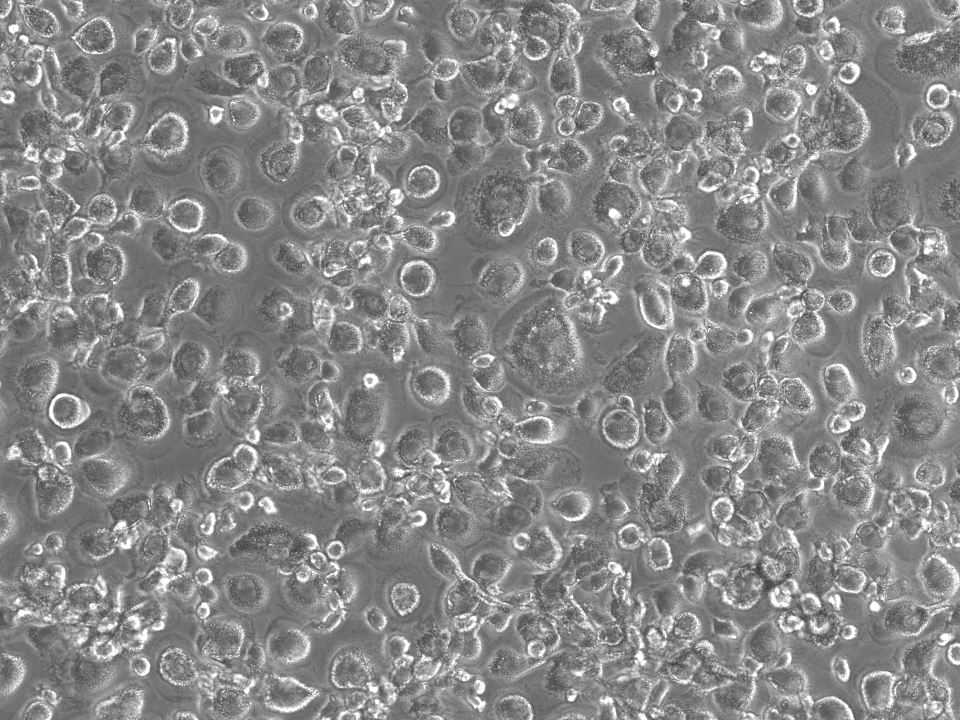

Supplement: Supplementary file 6 — Source data Fig. 4 [file 44319_2024_171_MOESM6_ESM.zip › Figure 4C/Fig. 4C_bottom, right.tif]

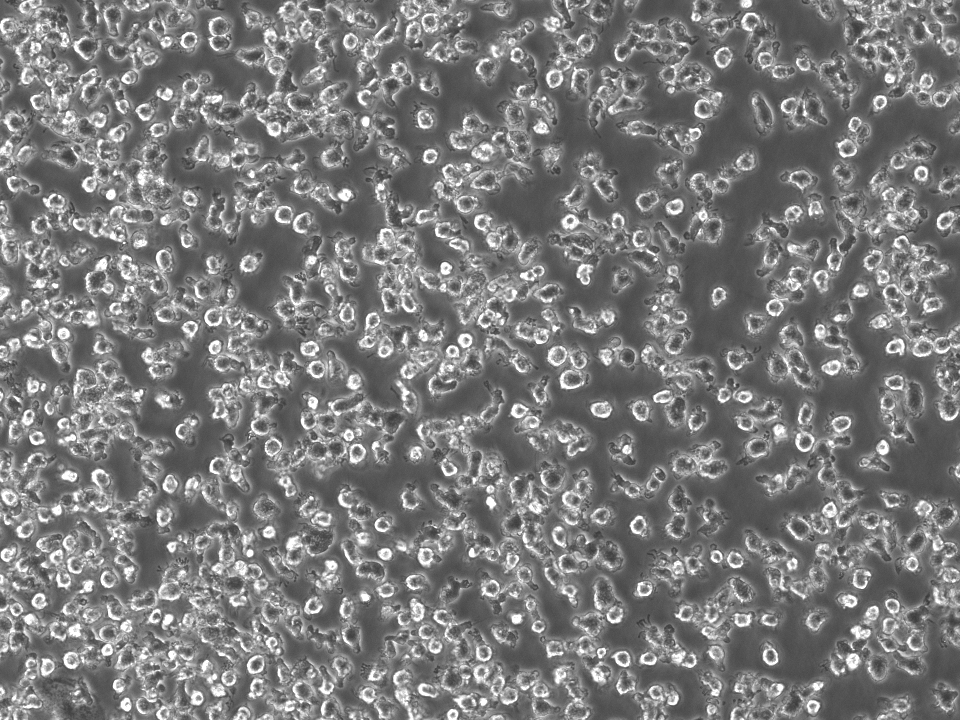

Supplement: Supplementary file 6 — Source data Fig. 4 [file 44319_2024_171_MOESM6_ESM.zip › Figure 4C/Fig. 4C_upper left, replicates/Fig. 4C_upper left, replicate 2.tif]

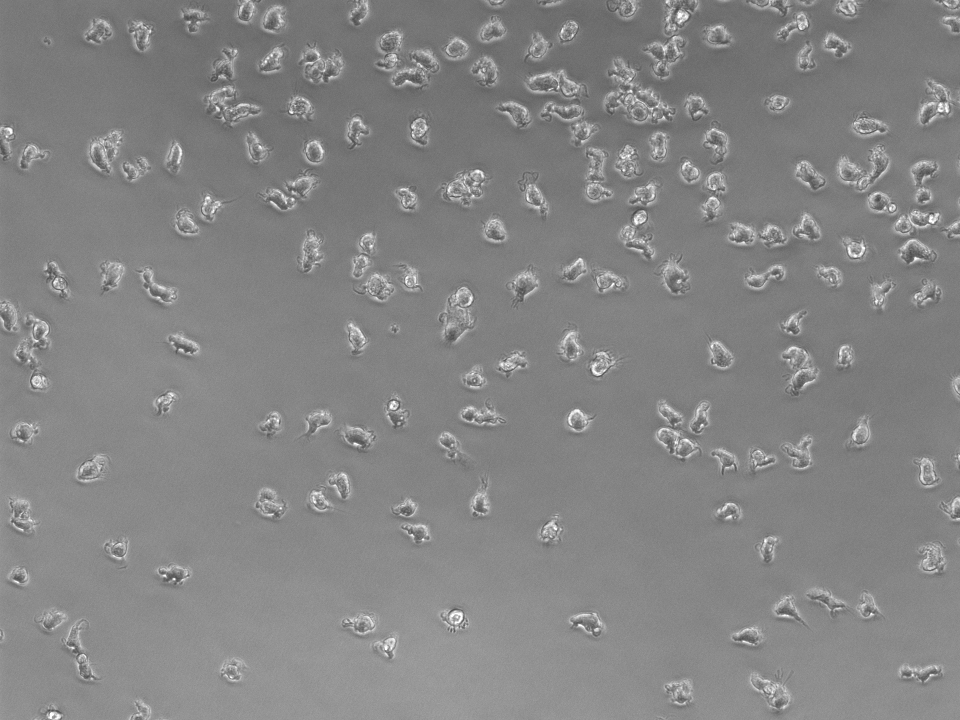

Supplement: Supplementary file 6 — Source data Fig. 4 [file 44319_2024_171_MOESM6_ESM.zip › Figure 4C/Fig. 4C_upper left, replicates/Fig. 4C_upper left, replicate 3.tif]

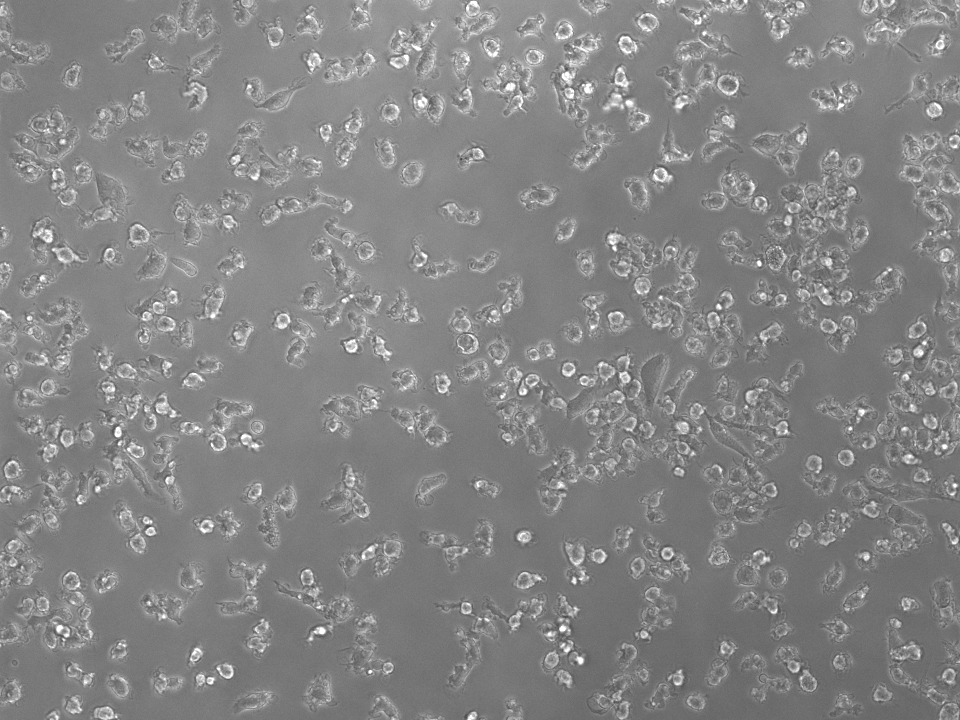

Supplement: Supplementary file 6 — Source data Fig. 4 [file 44319_2024_171_MOESM6_ESM.zip › Figure 4C/Fig. 4C_upper left, replicates/Fig. 4C_upper left, replicate 4.tif]

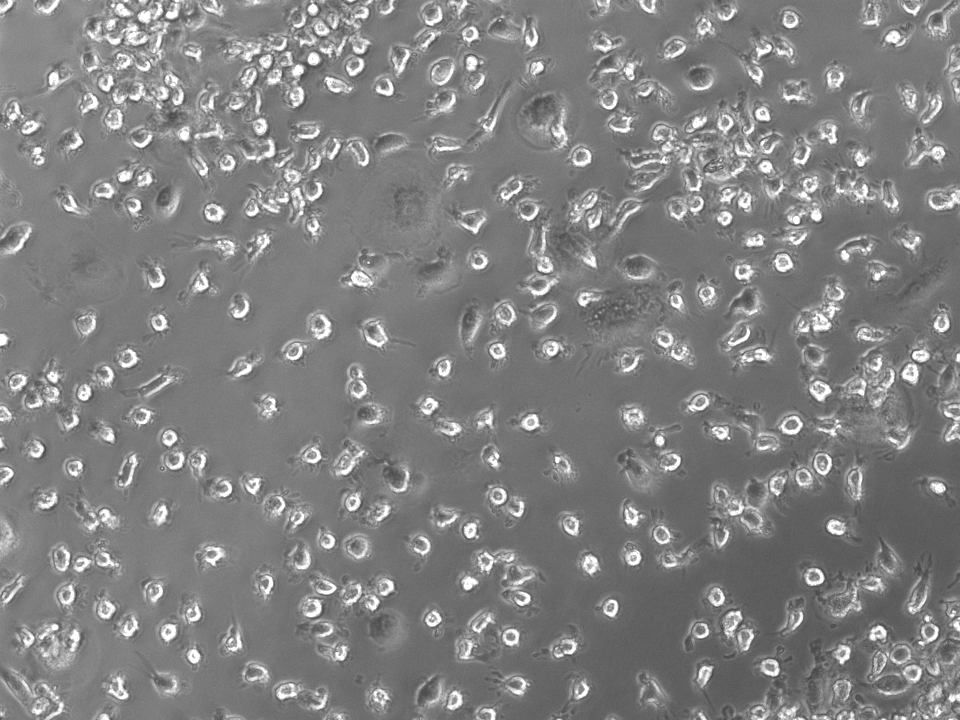

Supplement: Supplementary file 6 — Source data Fig. 4 [file 44319_2024_171_MOESM6_ESM.zip › Figure 4C/Fig. 4C_upper left, replicates/Fig. 4C_upper left, replicates 5.tif]

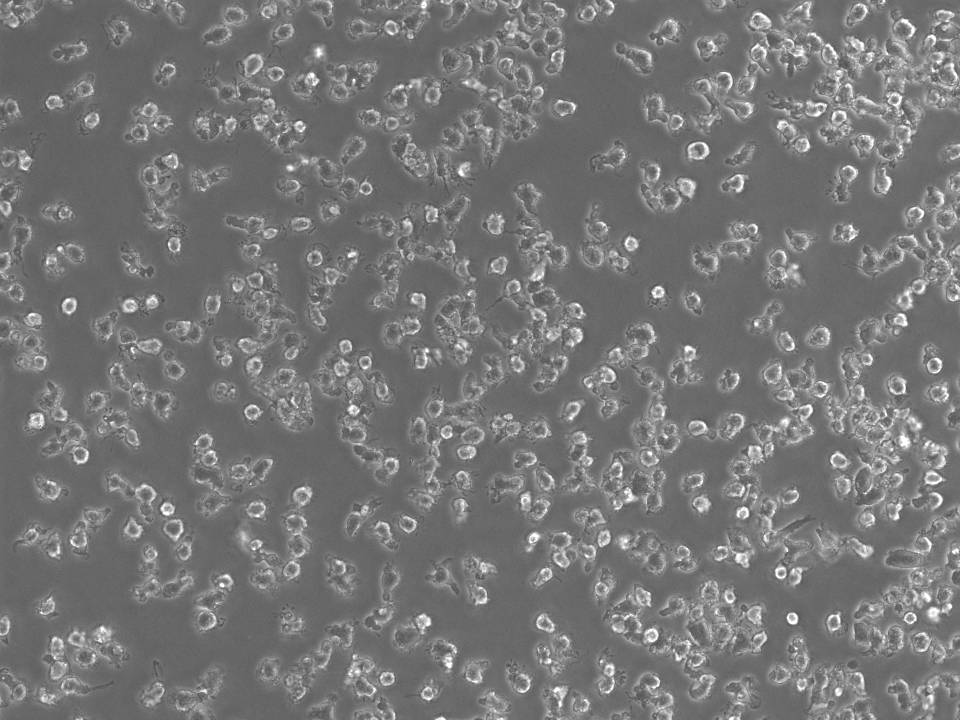

Supplement: Supplementary file 6 — Source data Fig. 4 [file 44319_2024_171_MOESM6_ESM.zip › Figure 4C/Fig. 4C_upper left.tif]

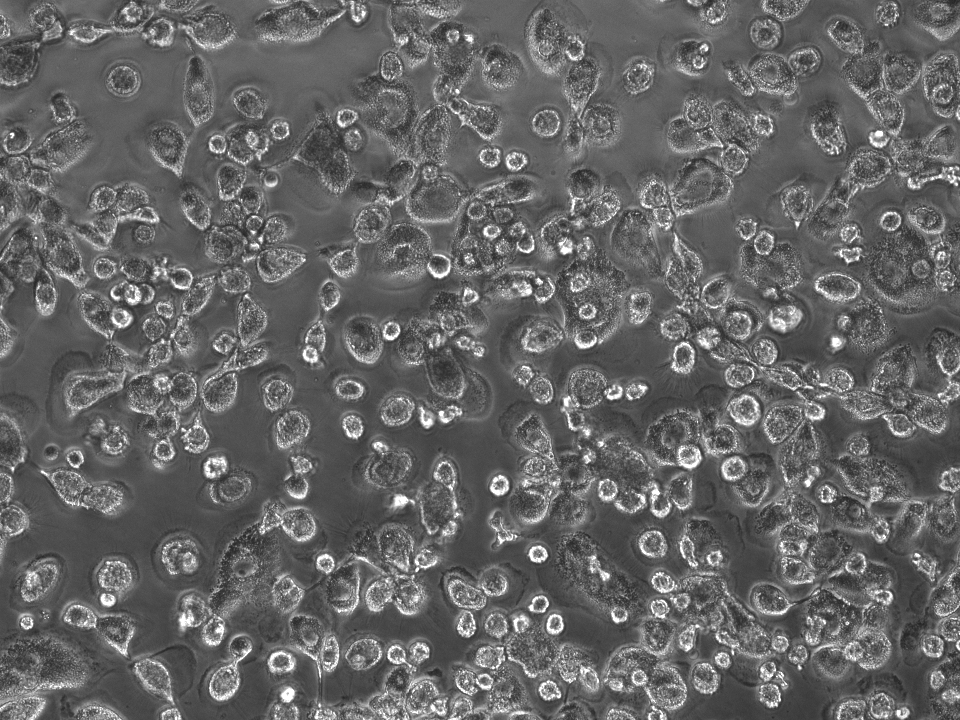

Supplement: Supplementary file 6 — Source data Fig. 4 [file 44319_2024_171_MOESM6_ESM.zip › Figure 4C/Fig. 4C_upper right, replicates/Fig. 4C_upper right, replicate 2.tif]

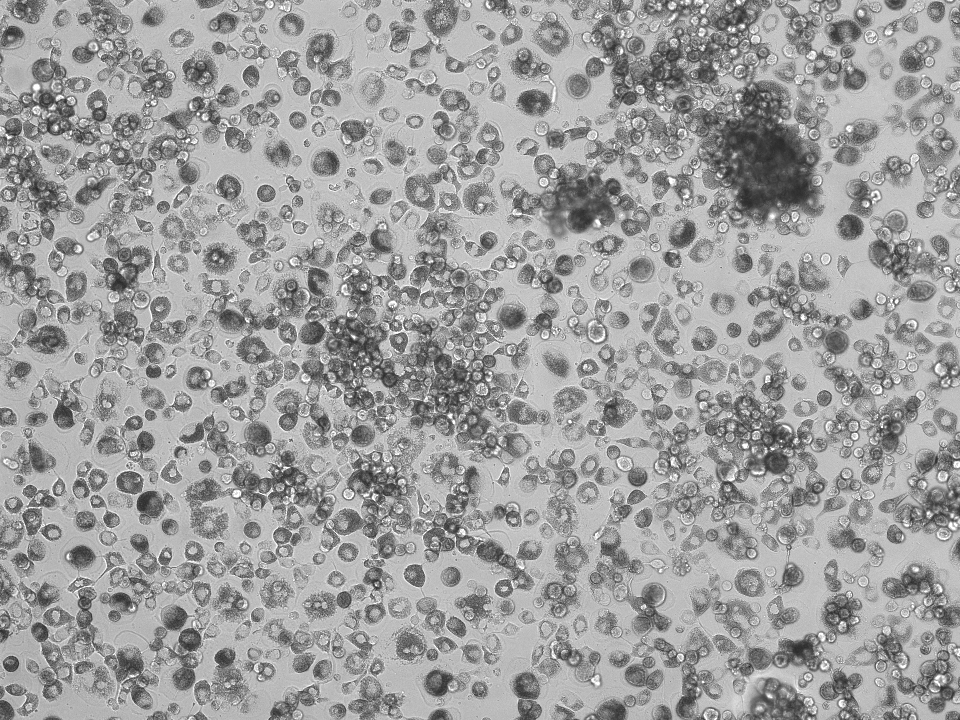

Supplement: Supplementary file 6 — Source data Fig. 4 [file 44319_2024_171_MOESM6_ESM.zip › Figure 4C/Fig. 4C_upper right, replicates/Fig. 4C_upper right, replicate 3.tif]

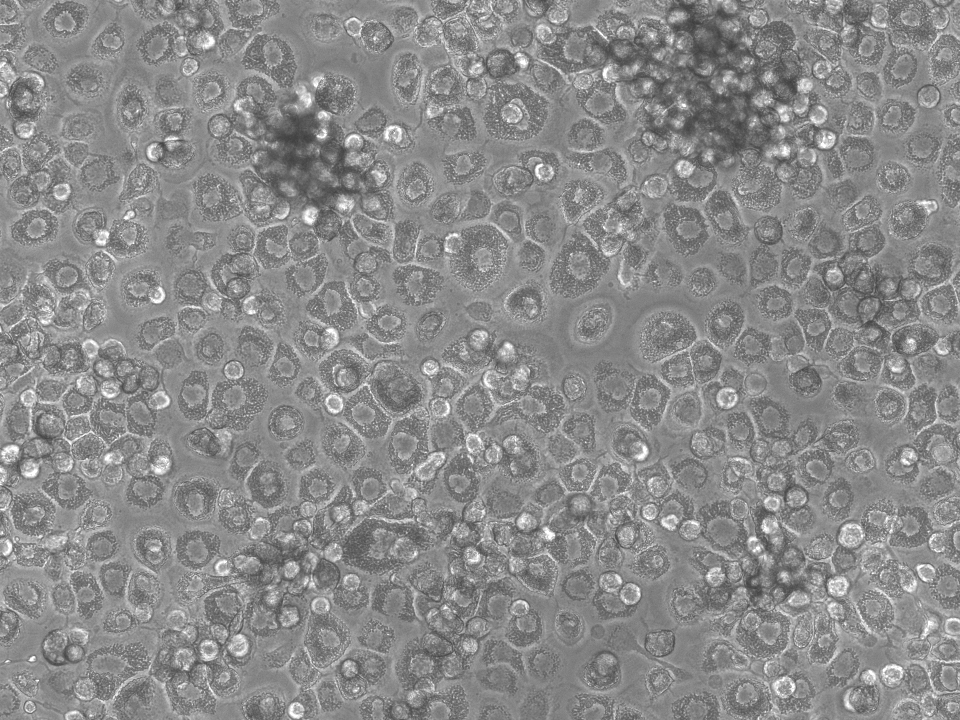

Supplement: Supplementary file 6 — Source data Fig. 4 [file 44319_2024_171_MOESM6_ESM.zip › Figure 4C/Fig. 4C_upper right.tif]

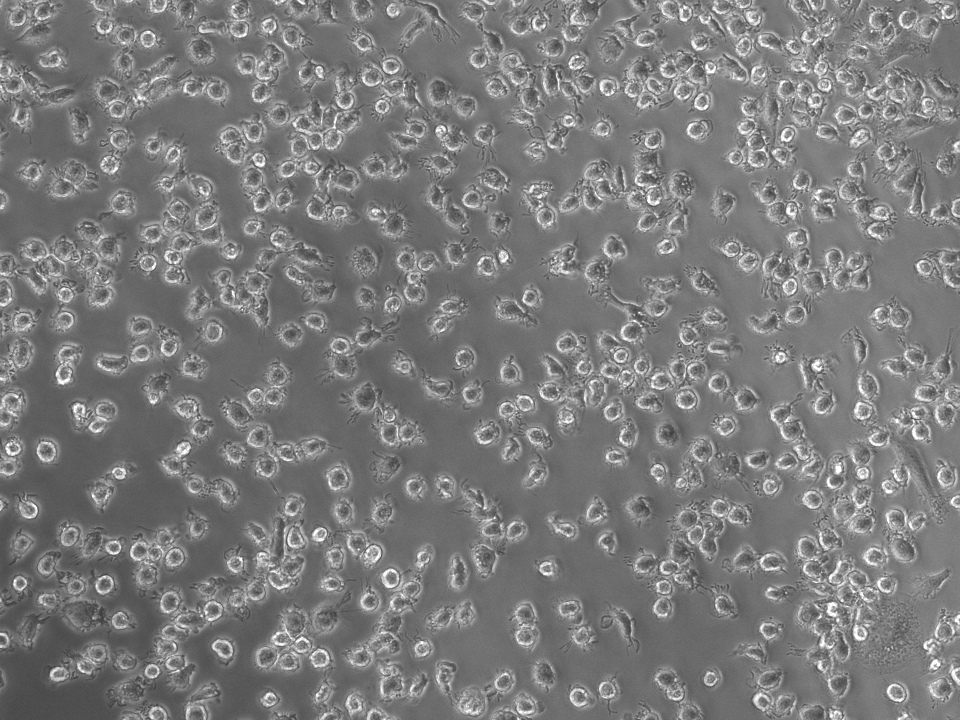

Supplement: Supplementary file 8 — Source data Fig. 6 [file 44319_2024_171_MOESM8_ESM.zip › Figure 6E/Fig. 6E_MOI=0_0 pg_mL IFNb.tif]

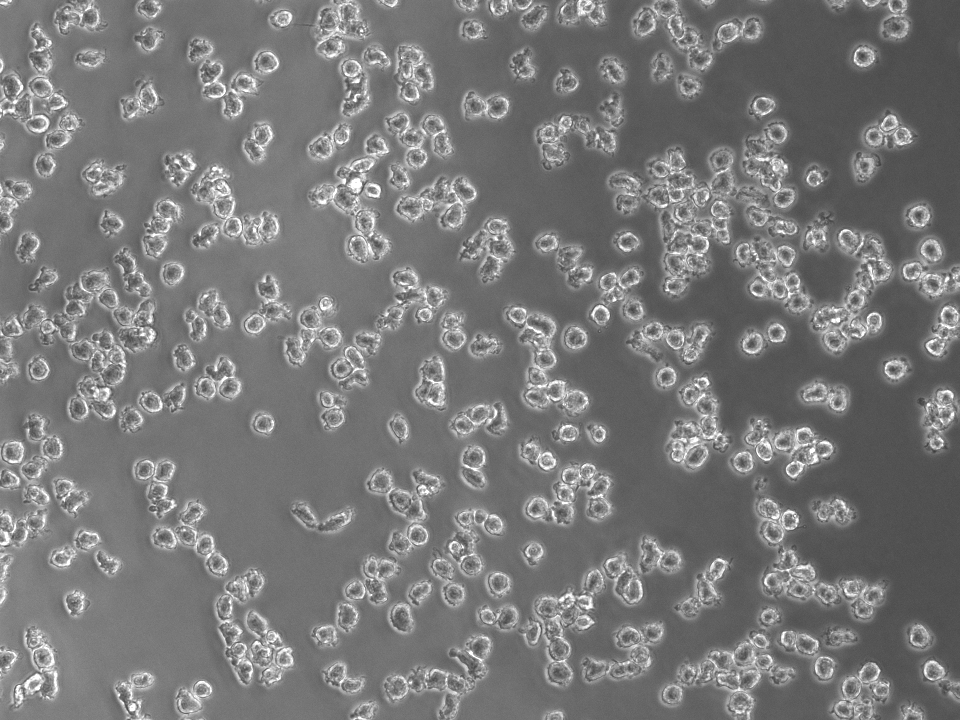

Supplement: Supplementary file 8 — Source data Fig. 6 [file 44319_2024_171_MOESM8_ESM.zip › Figure 6E/Fig. 6E_MOI=0_1250 pg_mL IFNb.tif]

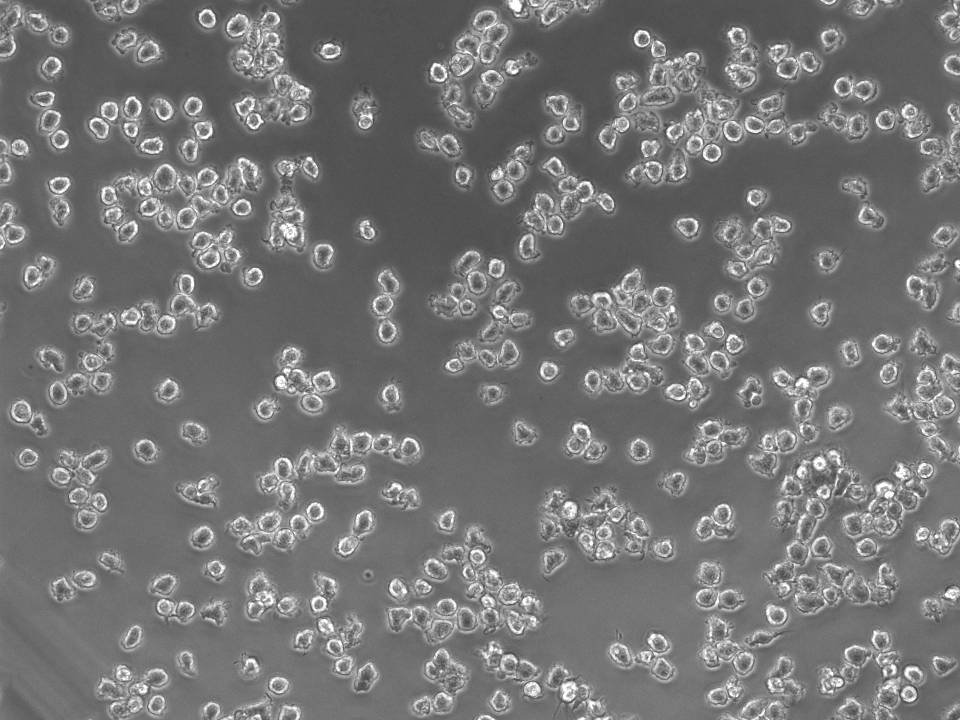

Supplement: Supplementary file 8 — Source data Fig. 6 [file 44319_2024_171_MOESM8_ESM.zip › Figure 6E/Fig. 6E_MOI=0_250 pg_mL IFNb.tif]

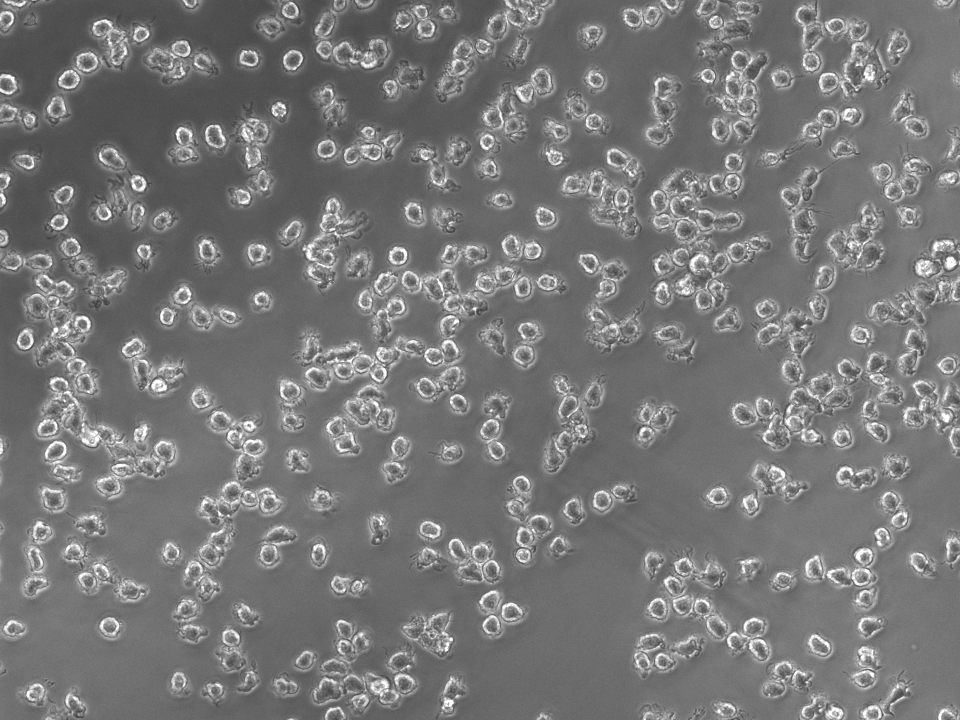

Supplement: Supplementary file 8 — Source data Fig. 6 [file 44319_2024_171_MOESM8_ESM.zip › Figure 6E/Fig. 6E_MOI=0_50 pg_mL IFNb.tif]

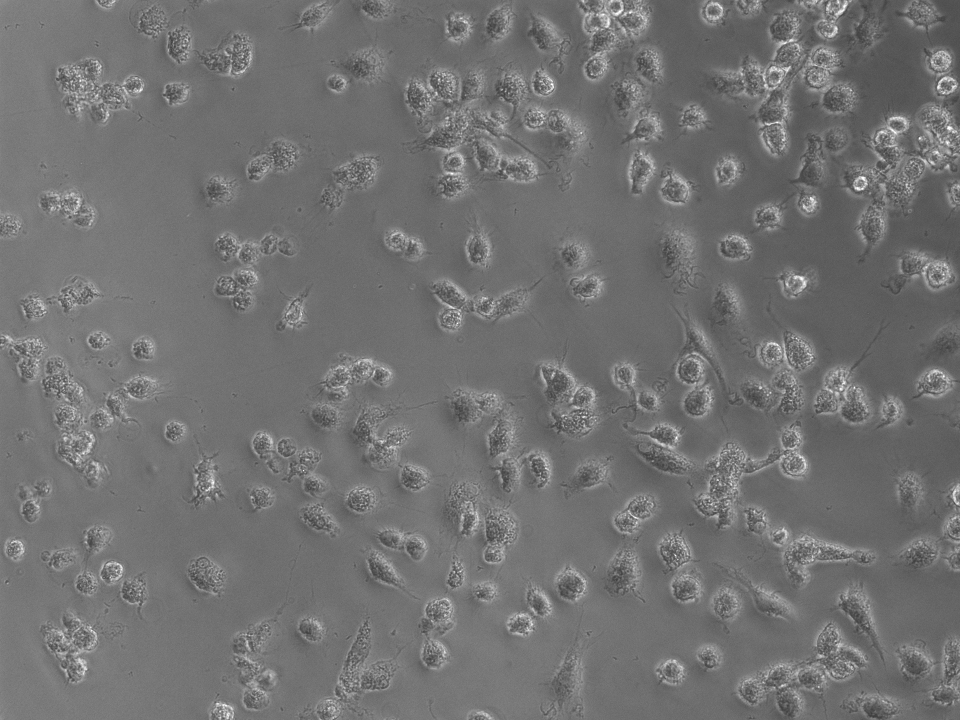

Supplement: Supplementary file 8 — Source data Fig. 6 [file 44319_2024_171_MOESM8_ESM.zip › Figure 6E/Fig. 6E_MOI=5_0 pg_mL IFNb.tif]

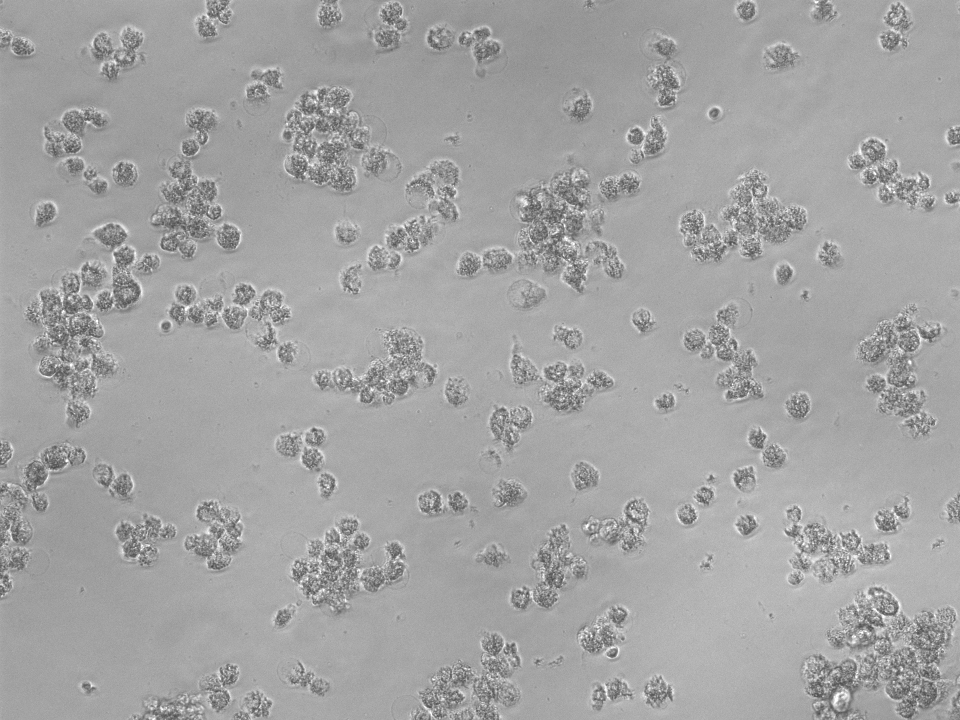

Supplement: Supplementary file 8 — Source data Fig. 6 [file 44319_2024_171_MOESM8_ESM.zip › Figure 6E/Fig. 6E_MOI=5_1250 pg_mL IFNb.tif]

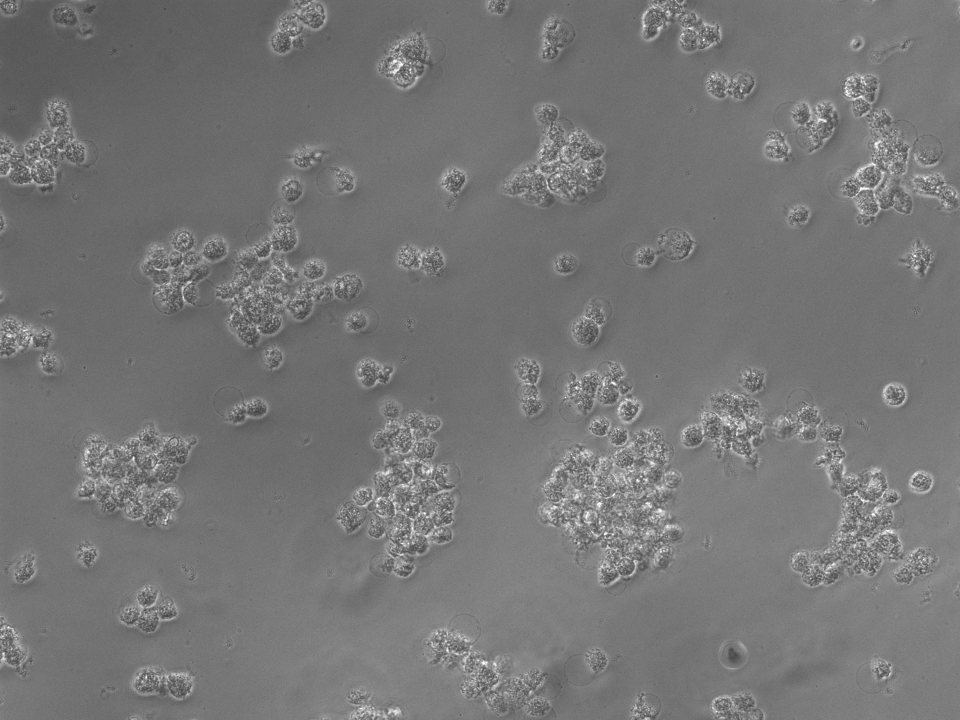

Supplement: Supplementary file 8 — Source data Fig. 6 [file 44319_2024_171_MOESM8_ESM.zip › Figure 6E/Fig. 6E_MOI=5_250 pg_mL IFNb.tif]

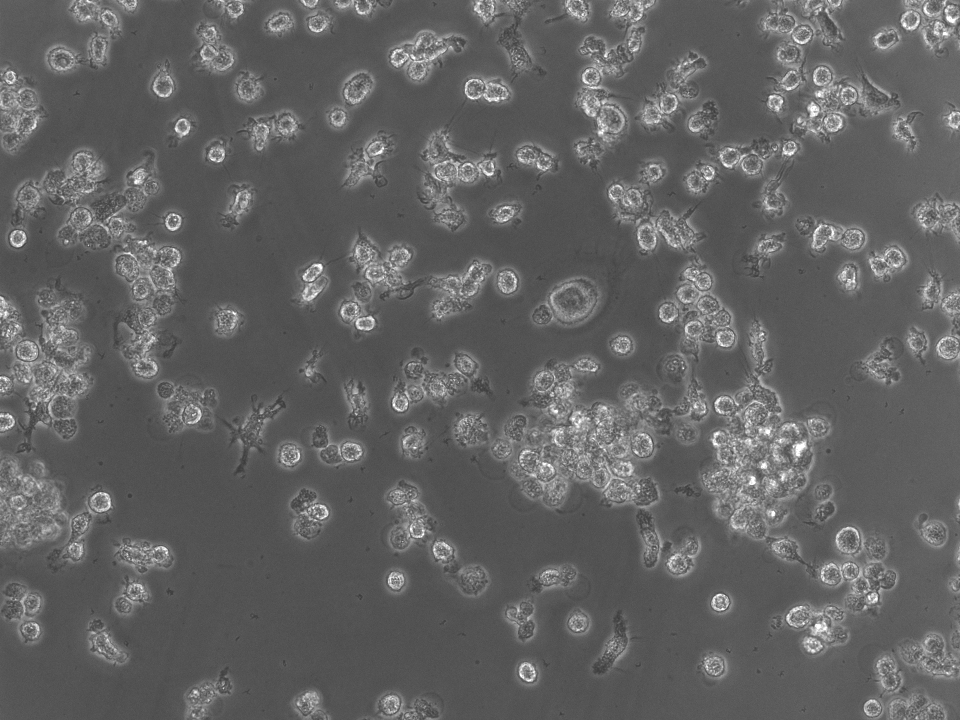

Supplement: Supplementary file 8 — Source data Fig. 6 [file 44319_2024_171_MOESM8_ESM.zip › Figure 6E/Fig. 6E_MOI=5_50 pg_mL IFNb.tif]

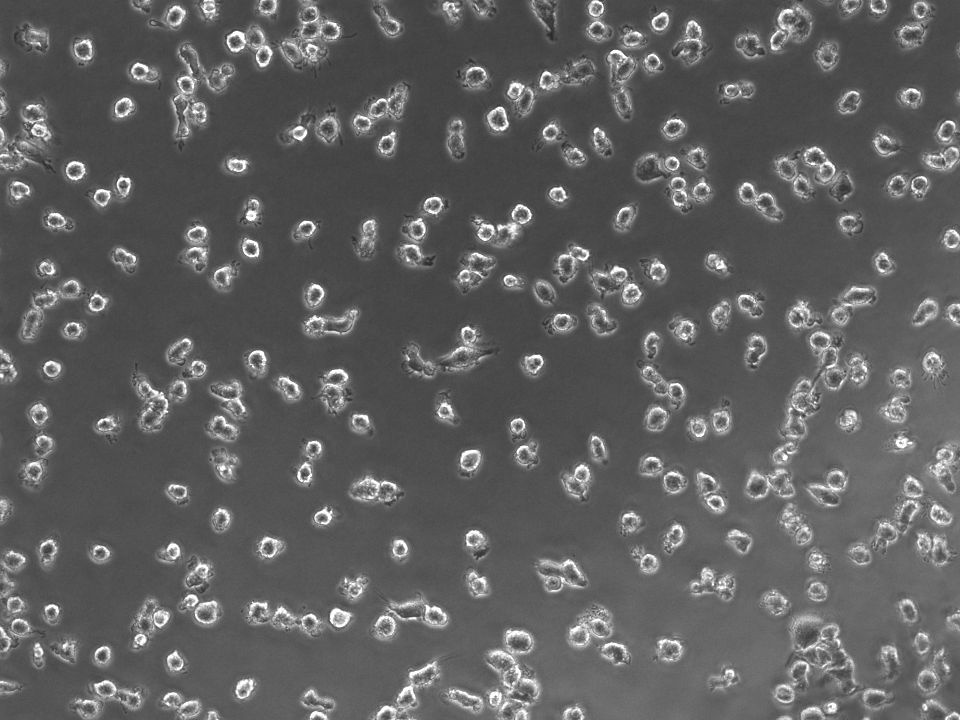

Supplement: Supplementary file 8 — Source data Fig. 6 [file 44319_2024_171_MOESM8_ESM.zip › Figure 6E/Fig. 6E_replicates/Fig.6E_rep 2_MOI=0_0 pg_mL IFNb_09.tif]

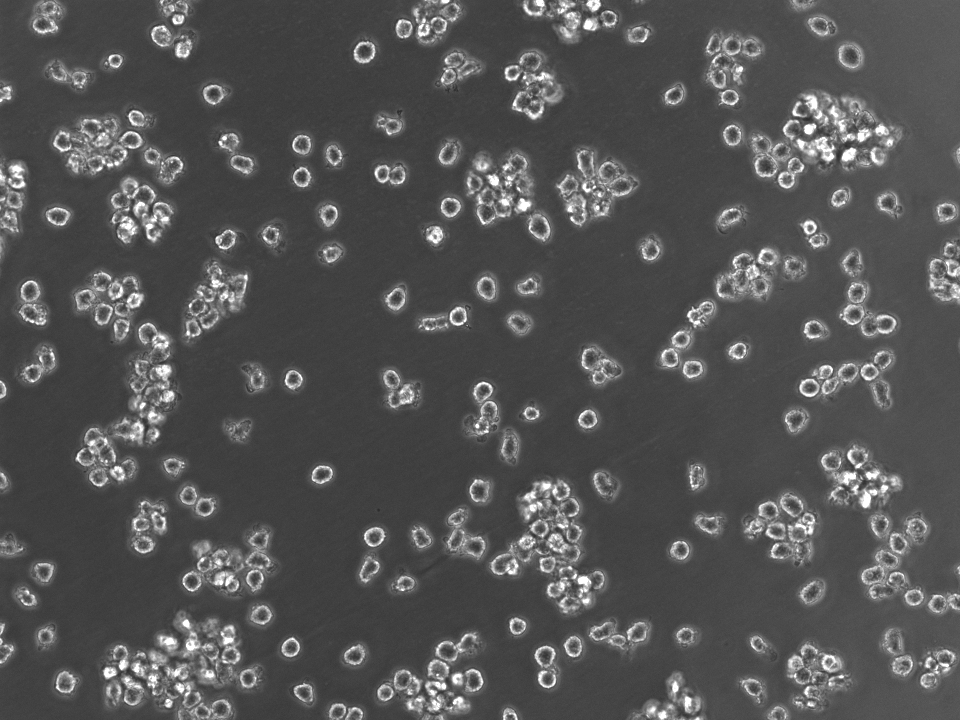

Supplement: Supplementary file 8 — Source data Fig. 6 [file 44319_2024_171_MOESM8_ESM.zip › Figure 6E/Fig. 6E_replicates/Fig.6E_rep 2_MOI=0_1250 pg_mL IFNb.tif]

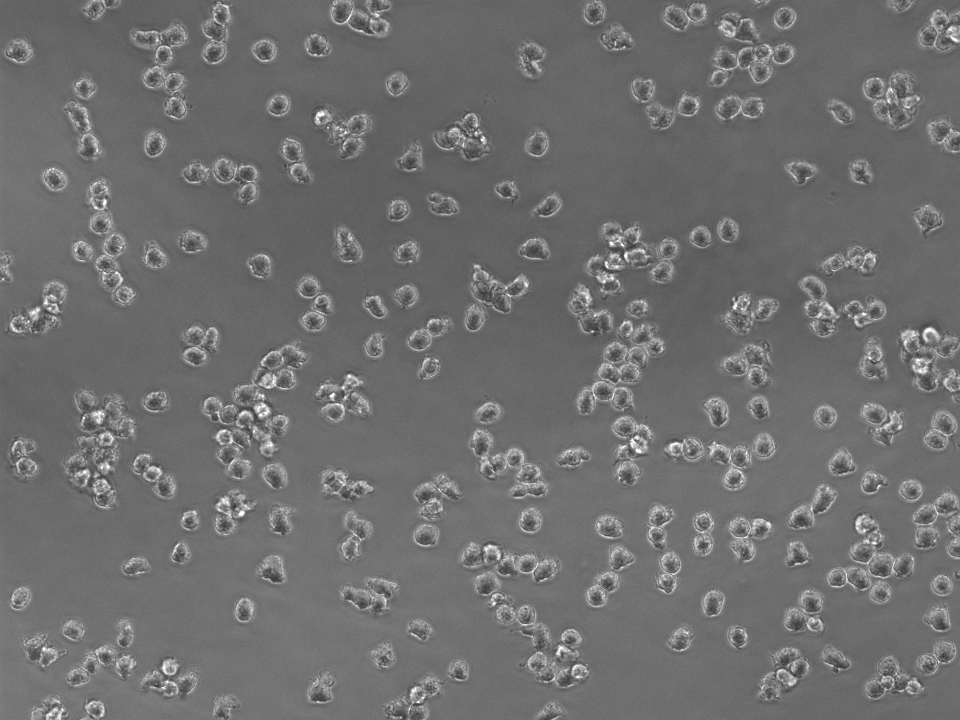

Supplement: Supplementary file 8 — Source data Fig. 6 [file 44319_2024_171_MOESM8_ESM.zip › Figure 6E/Fig. 6E_replicates/Fig.6E_rep 2_MOI=0_250 pg_mL IFNbtif.tif]

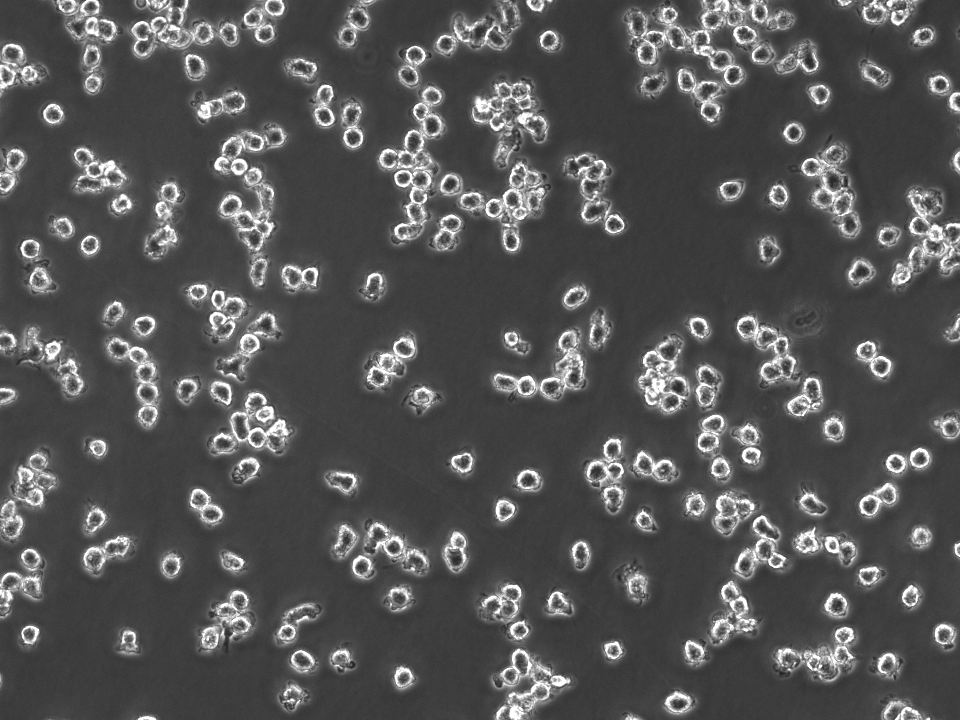

Supplement: Supplementary file 8 — Source data Fig. 6 [file 44319_2024_171_MOESM8_ESM.zip › Figure 6E/Fig. 6E_replicates/Fig.6E_rep 2_MOI=0_50 pg_mL IFNb.tif]

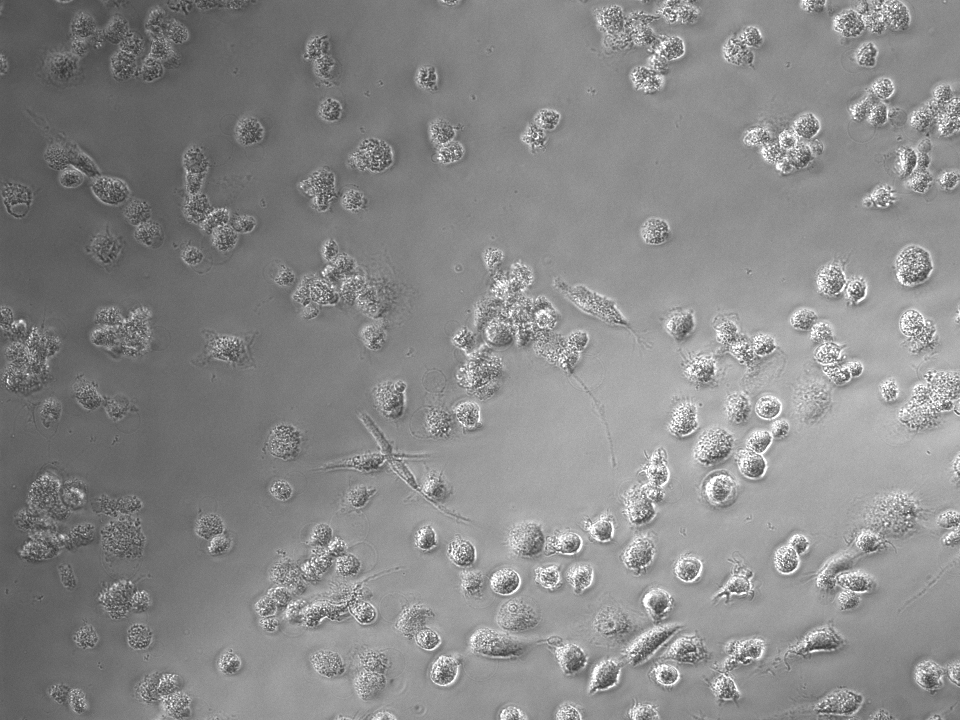

Supplement: Supplementary file 8 — Source data Fig. 6 [file 44319_2024_171_MOESM8_ESM.zip › Figure 6E/Fig. 6E_replicates/Fig.6E_rep 2_MOI=5_0 pg_mL IFNb.tif]

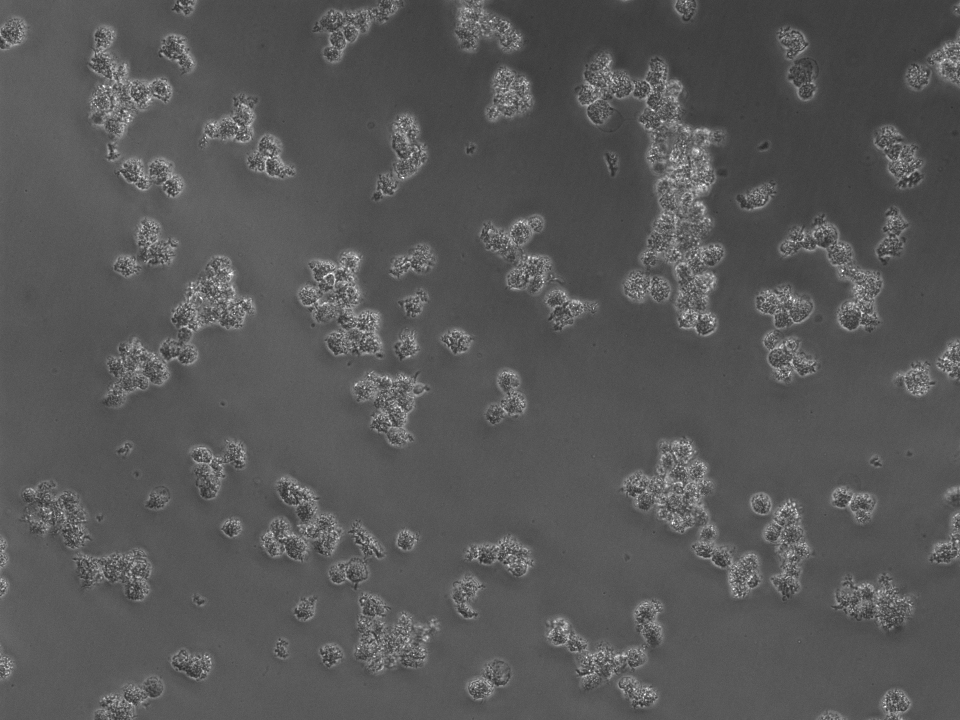

Supplement: Supplementary file 8 — Source data Fig. 6 [file 44319_2024_171_MOESM8_ESM.zip › Figure 6E/Fig. 6E_replicates/Fig.6E_rep 2_MOI=5_1250 pg_mL IFNb.tif]

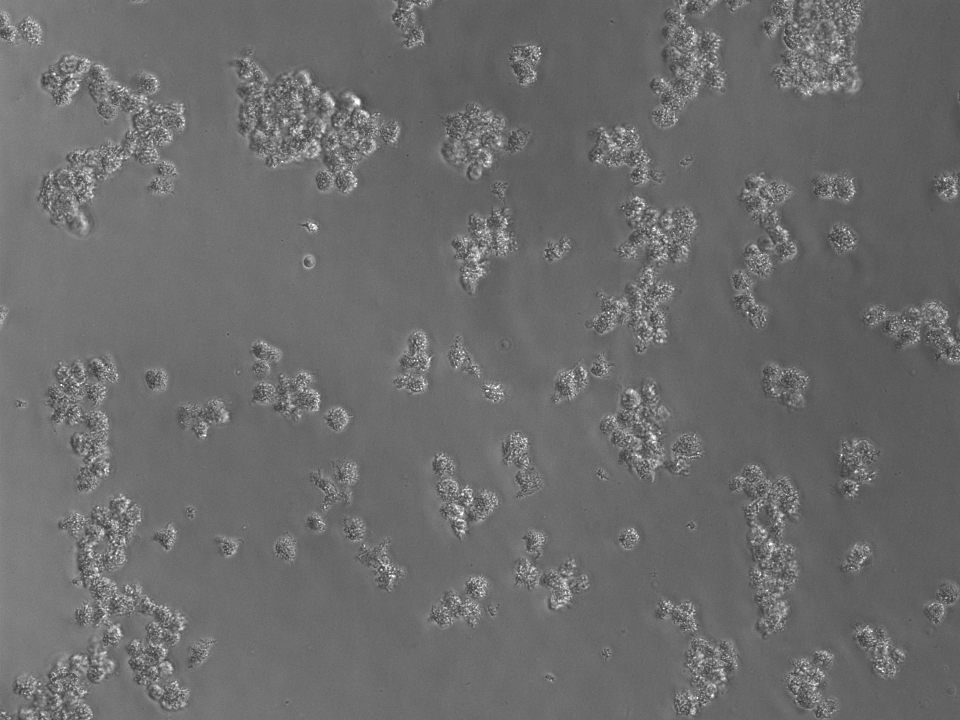

Supplement: Supplementary file 8 — Source data Fig. 6 [file 44319_2024_171_MOESM8_ESM.zip › Figure 6E/Fig. 6E_replicates/Fig.6E_rep 2_MOI=5_250 pg_mL IFNb.tif]

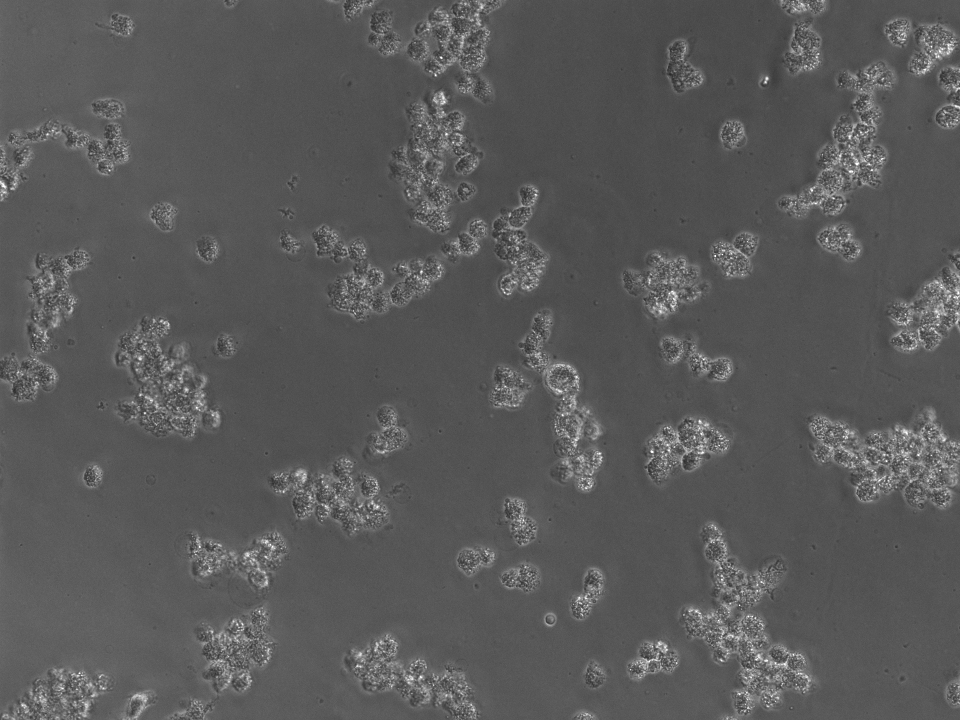

Supplement: Supplementary file 8 — Source data Fig. 6 [file 44319_2024_171_MOESM8_ESM.zip › Figure 6E/Fig. 6E_replicates/Fig.6E_rep 2_MOI=5_50 pg_mL IFNb.tif]

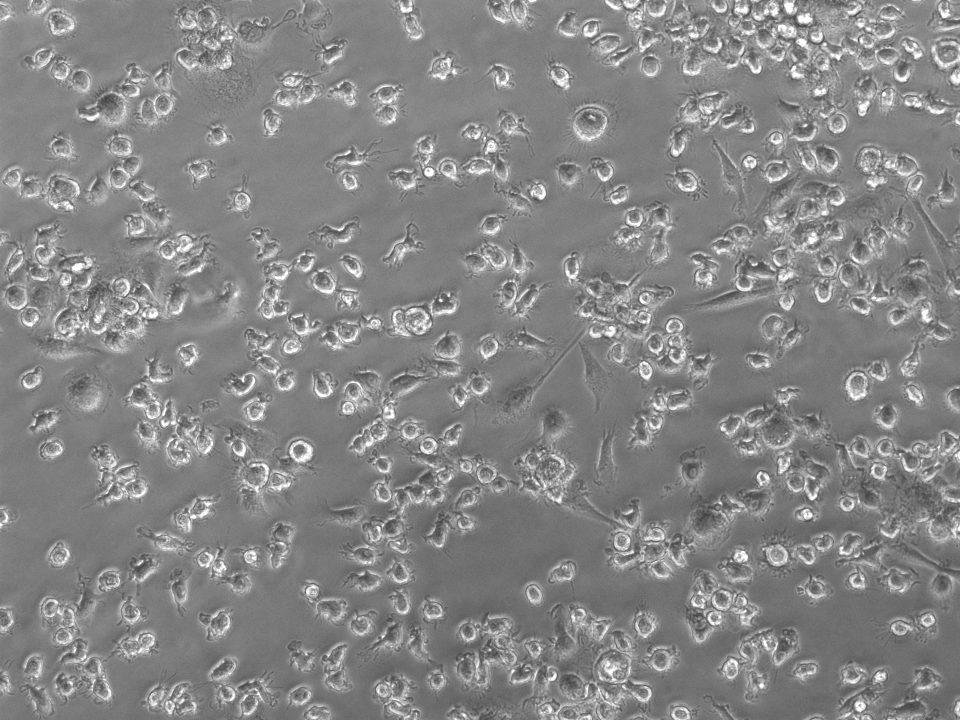

Supplement: Supplementary file 8 — Source data Fig. 6 [file 44319_2024_171_MOESM8_ESM.zip › Figure 6E/Fig. 6E_replicates/Fig.6E_rep 3_MOI=0_0 pg_mL IFNb.tif]

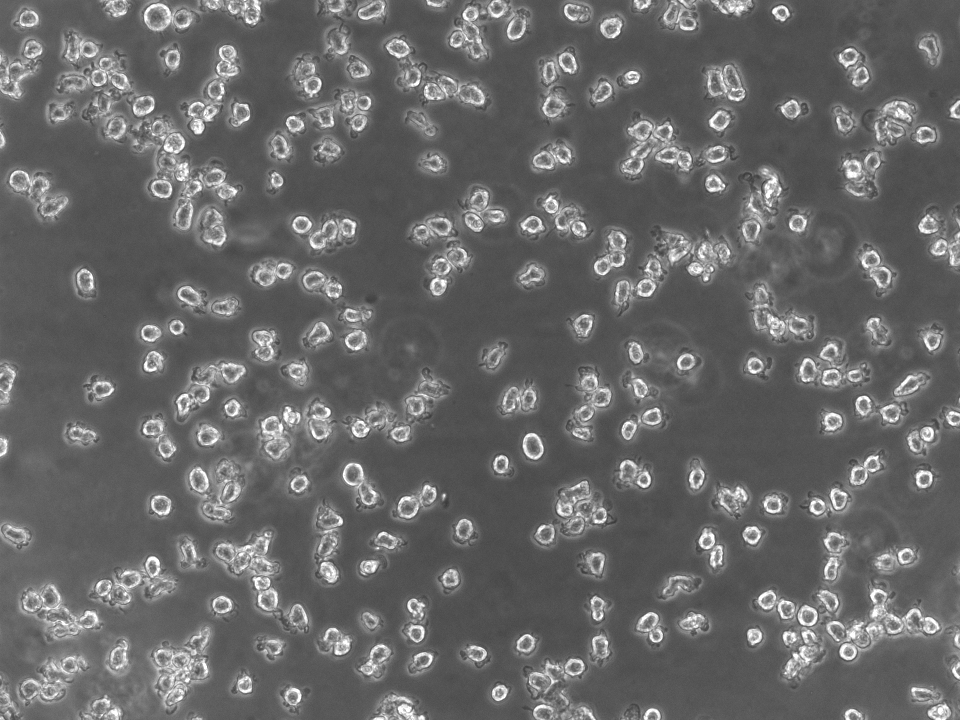

Supplement: Supplementary file 8 — Source data Fig. 6 [file 44319_2024_171_MOESM8_ESM.zip › Figure 6E/Fig. 6E_replicates/Fig.6E_rep 3_MOI=0_1250 pg_mL IFNb.tif]

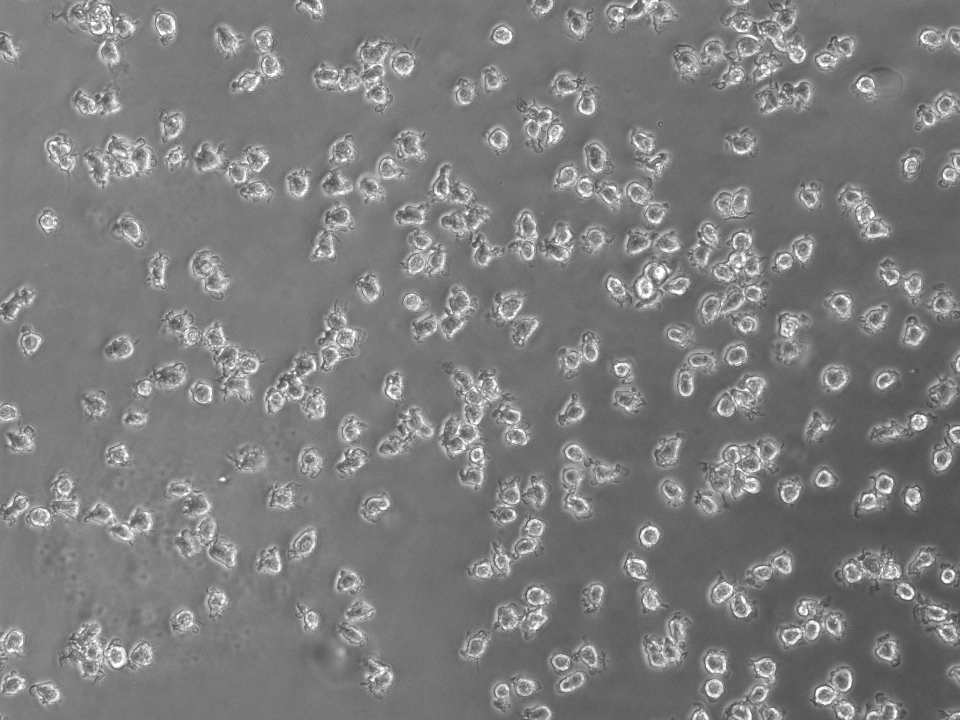

Supplement: Supplementary file 8 — Source data Fig. 6 [file 44319_2024_171_MOESM8_ESM.zip › Figure 6E/Fig. 6E_replicates/Fig.6E_rep 3_MOI=0_250 pg_mL IFNb.tif]

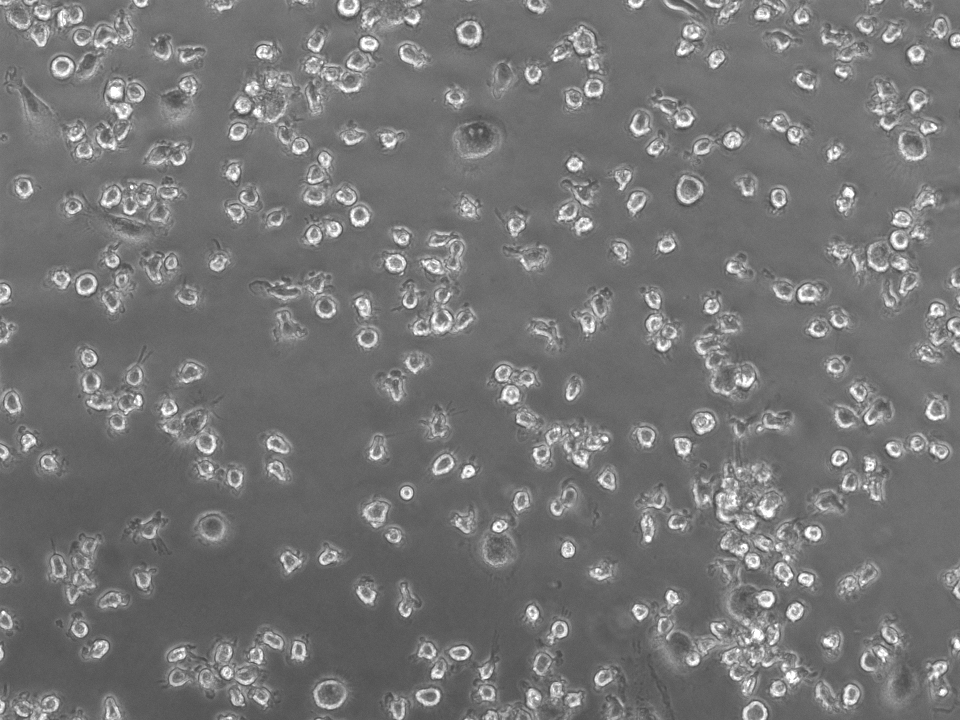

Supplement: Supplementary file 8 — Source data Fig. 6 [file 44319_2024_171_MOESM8_ESM.zip › Figure 6E/Fig. 6E_replicates/Fig.6E_rep 3_MOI=0_50 pg_mL IFNb .tif]

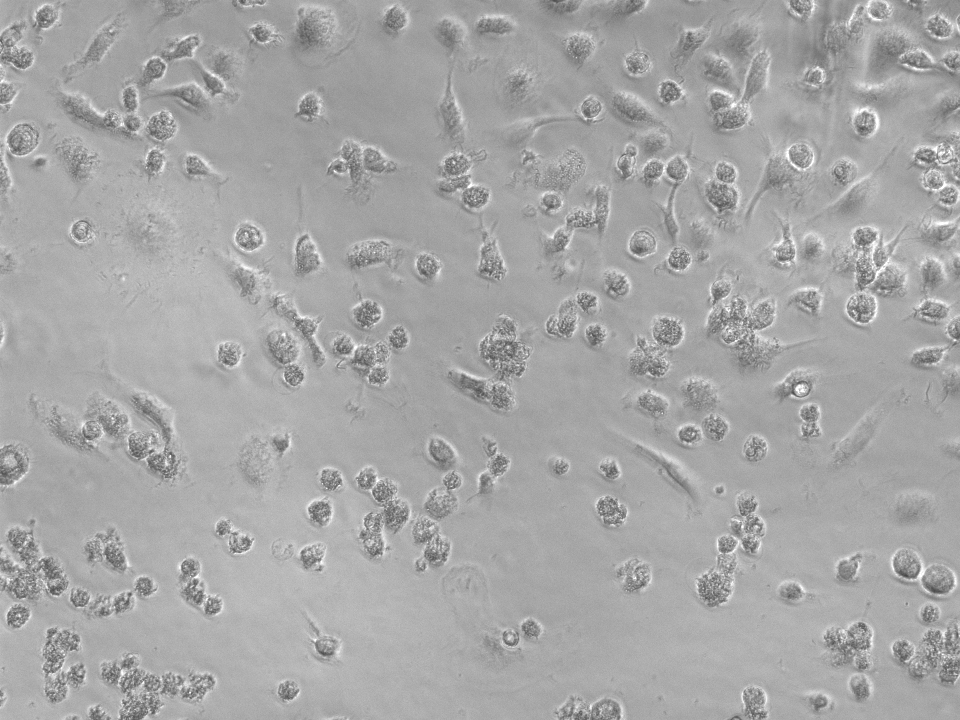

Supplement: Supplementary file 8 — Source data Fig. 6 [file 44319_2024_171_MOESM8_ESM.zip › Figure 6E/Fig. 6E_replicates/Fig.6E_rep 3_MOI=5_0 pg_mL IFNb.tif]

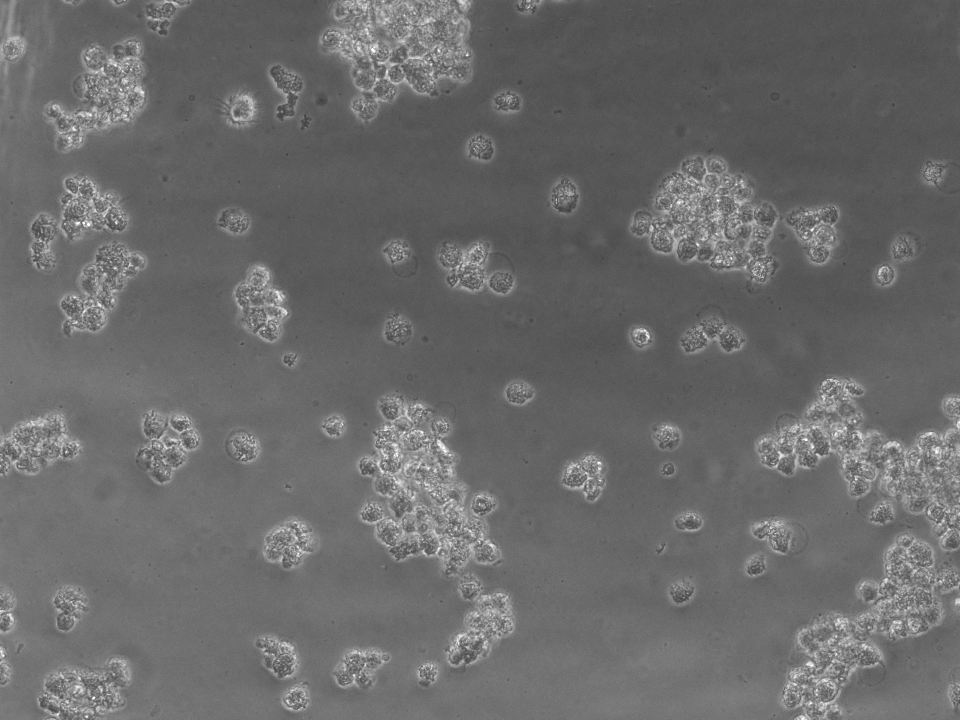

Supplement: Supplementary file 8 — Source data Fig. 6 [file 44319_2024_171_MOESM8_ESM.zip › Figure 6E/Fig. 6E_replicates/Fig.6E_rep 3_MOI=5_1250 pg_mL IFNb.tif]

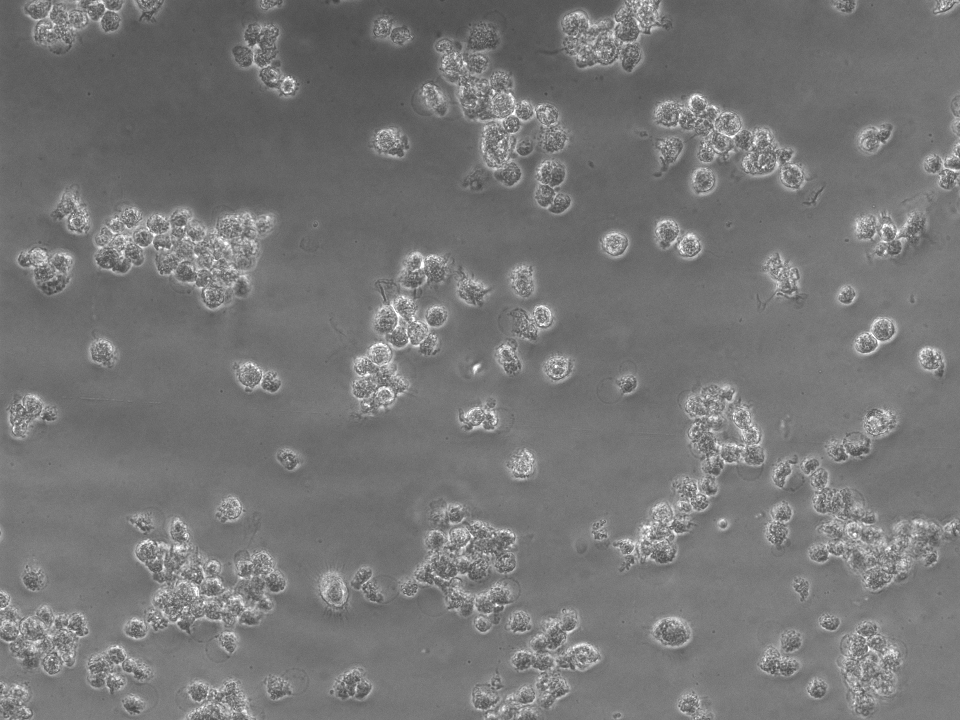

Supplement: Supplementary file 8 — Source data Fig. 6 [file 44319_2024_171_MOESM8_ESM.zip › Figure 6E/Fig. 6E_replicates/Fig.6E_rep 3_MOI=5_250 pg_mL IFNb.tif]

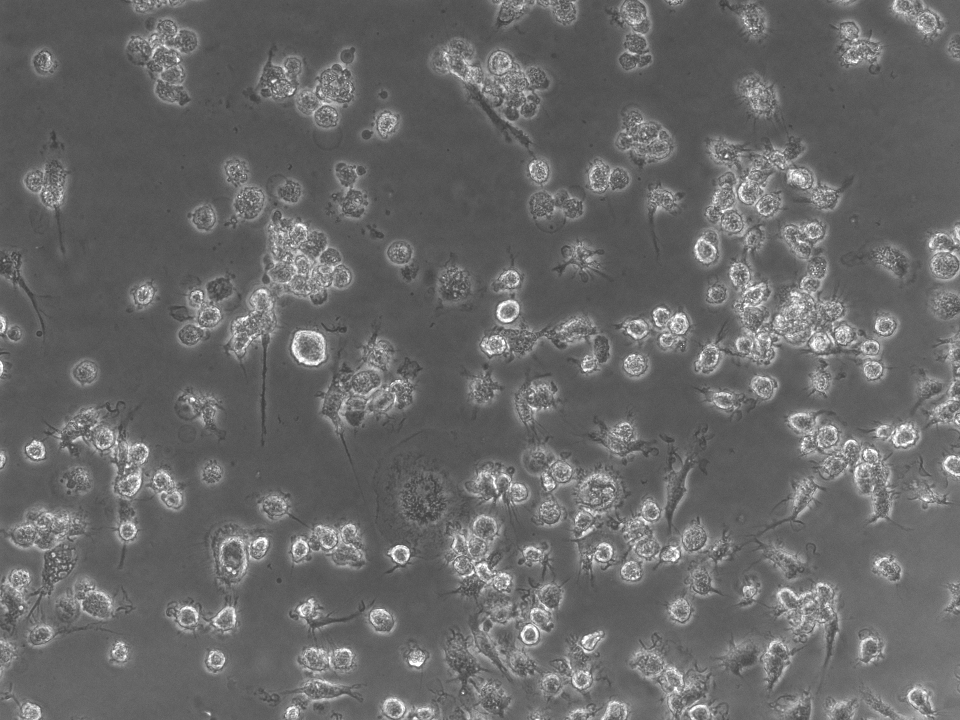

Supplement: Supplementary file 8 — Source data Fig. 6 [file 44319_2024_171_MOESM8_ESM.zip › Figure 6E/Fig. 6E_replicates/Fig.6E_rep 3_MOI=5_50 pg_mL IFNb.tif]
