# Supplementary material for: Type I interferon exacerbates Mycobacterium tuberculosis induced human macrophage death
Source: EMBO Rep. 2024 Jun 12;25(7):15. doi: 10.1038/s44319-024-00171-0 (PMC11239827; doi:10.1038/s44319-024-00171-0)
Supplement: Supplementary file 9 — Expanded View Figures [file 44319_2024_171_MOESM9_ESM.pdf]

Expanded View Figures

Figure EV1. Neutralizing IFN-I Ab mix specifically ablates IFN-I signaling.

(**A**) MDM-2 ($n = 1$) were treated with anti-IFNAR1 mAb, anti-IFNAR2 mAb, IgG, or vehicle control (medium) for 2 h, then infected with tdTomato-Mtb. Images were taken at given timepoints post infection at 20X. Scale bars = 20 µm. (**B**, **C**) MDM-2 ($n = 1$) were treated with mAbs against IFNAR1, IFNAR2, IgG isotype control, or vehicle control (media) for 2 h, stimulated with IFN-b for 15–20 min, and then lysates were collected for western blot with indicated antibodies. (**D**) MDM-2 ($n = 1$) were treated with IFN-I neutralizing Ab mixture (see Methods), IgG, or vehicle control (media) for 2 h and stimulated with the indicated concentrations of IFN-b for 15–20 min. Lysates were then collected for western blot with indicated antibodies. (**E**) MDM-1 ($n = 1$) were treated with IFN-I neutralizing Ab mixture, IgG, or vehicle control (medium) for 2 h and stimulated with IFN-b, IFN-g, or IFN-l for 15–20 min. Lysates were then collected for western blot with indicated antibodies. (**F**) IFN-III HEKBlue 293 cells ($n = 1$) were treated with IFN-I neutralizing Ab mixture, IgG, or vehicle control (medium) for 2 h and then stimulated with 1000 pg/mL of IFN-l1 (IL-29) overnight. The level of IFN-III signaling was measured via QUANTI-Blue assay. The concentration of IFN-l was estimated by performing a sigmoidal, 4 parameter interpolation from an IL-29 standard curve, using Prism's software. Data Information: Each • represents the average of 3 technical replicates, while *n* indicates biological replicates.

 © The Author(s)

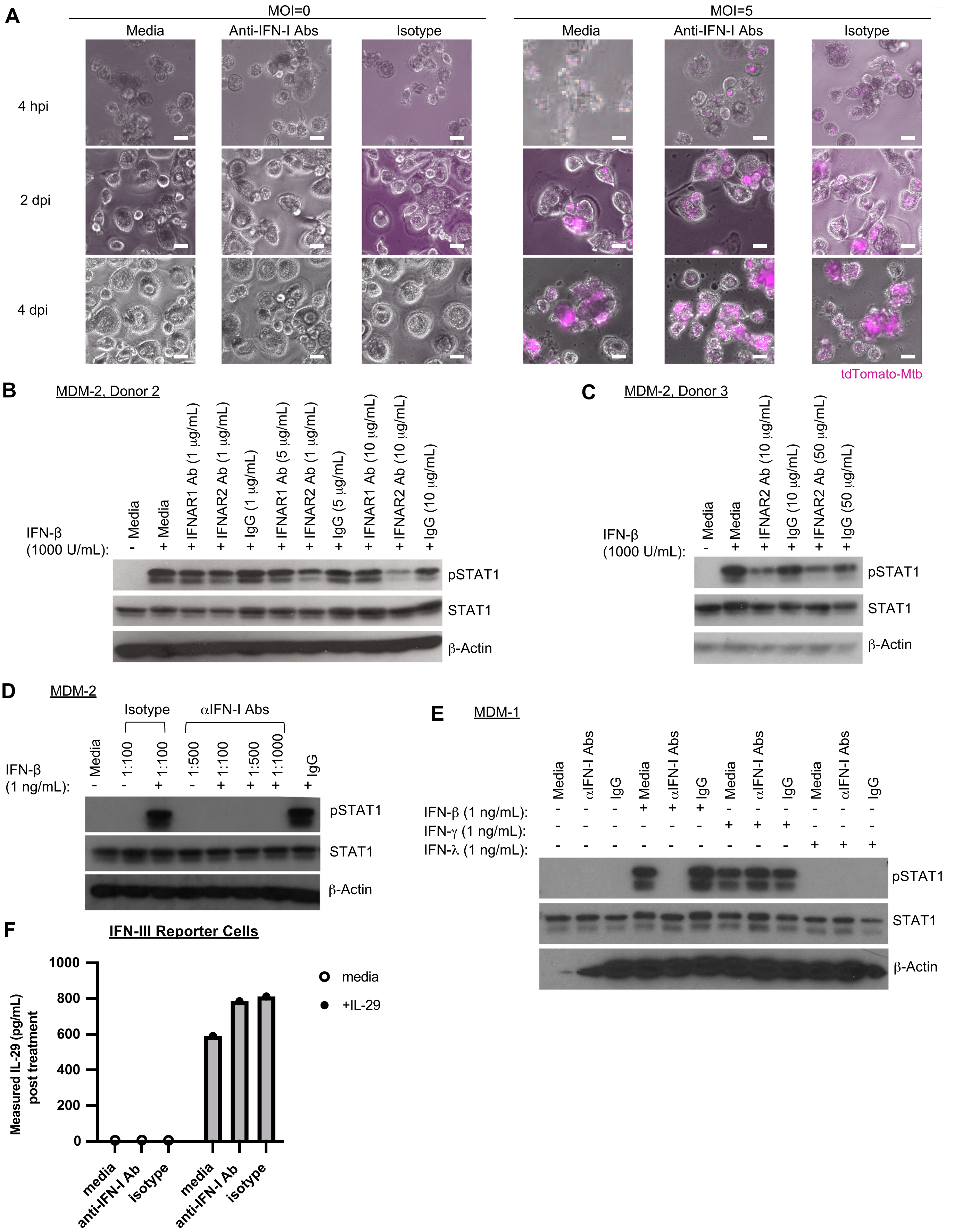
A
MOI=0
MOI=5
Media
Anti-IFN-I Abs
Isotype
4 hpi
2 dpi
4 dpi
tdTomato-Mtb
B
MDM-2, Donor 2
IFN-β (1000 U/mL):
- Media
+ Media
+ IFNAR1 Ab (1 μg/mL)
+ IFNAR2 Ab (1 μg/mL)
+ IgG (1 μg/mL)
+ IFNAR1 Ab (5 μg/mL)
+ IFNAR2 Ab (1 μg/mL)
+ IgG (5 μg/mL)
+ IFNAR1 Ab (10 μg/mL)
+ IFNAR2 Ab (10 μg/mL)
+ IgG (10 μg/mL)
pSTAT1
STAT1
β-Actin
C
MDM-2, Donor 3
IFN-β (1000 U/mL):
- Media
+ Media
+ IFNAR2 Ab (10 μg/mL)
+ IgG (10 μg/mL)
+ IFNAR2 Ab (50 μg/mL)
+ IgG (50 μg/mL)
pSTAT1
STAT1
β-Actin
D
MDM-2
Isotype
αIFN-I Abs
IFN-β (1 ng/mL):
- Media
- 1:100
+ 1:100
- 1:500
+ 1:100
+ 1:500
+ 1:1000
+ IgG
pSTAT1
STAT1
β-Actin
E
MDM-1
Media
αIFN-I Abs
IgG
IFN-β (1 ng/mL):
IFN-γ (1 ng/mL):
IFN-λ (1 ng/mL):
pSTAT1
STAT1
β-Actin
F
IFN-III Reporter Cells
Measured IL-29 (pg/mL) post treatment
1000
800
600
400
200
0
media
anti-IFN-I Ab
isotype
media
+IL-29

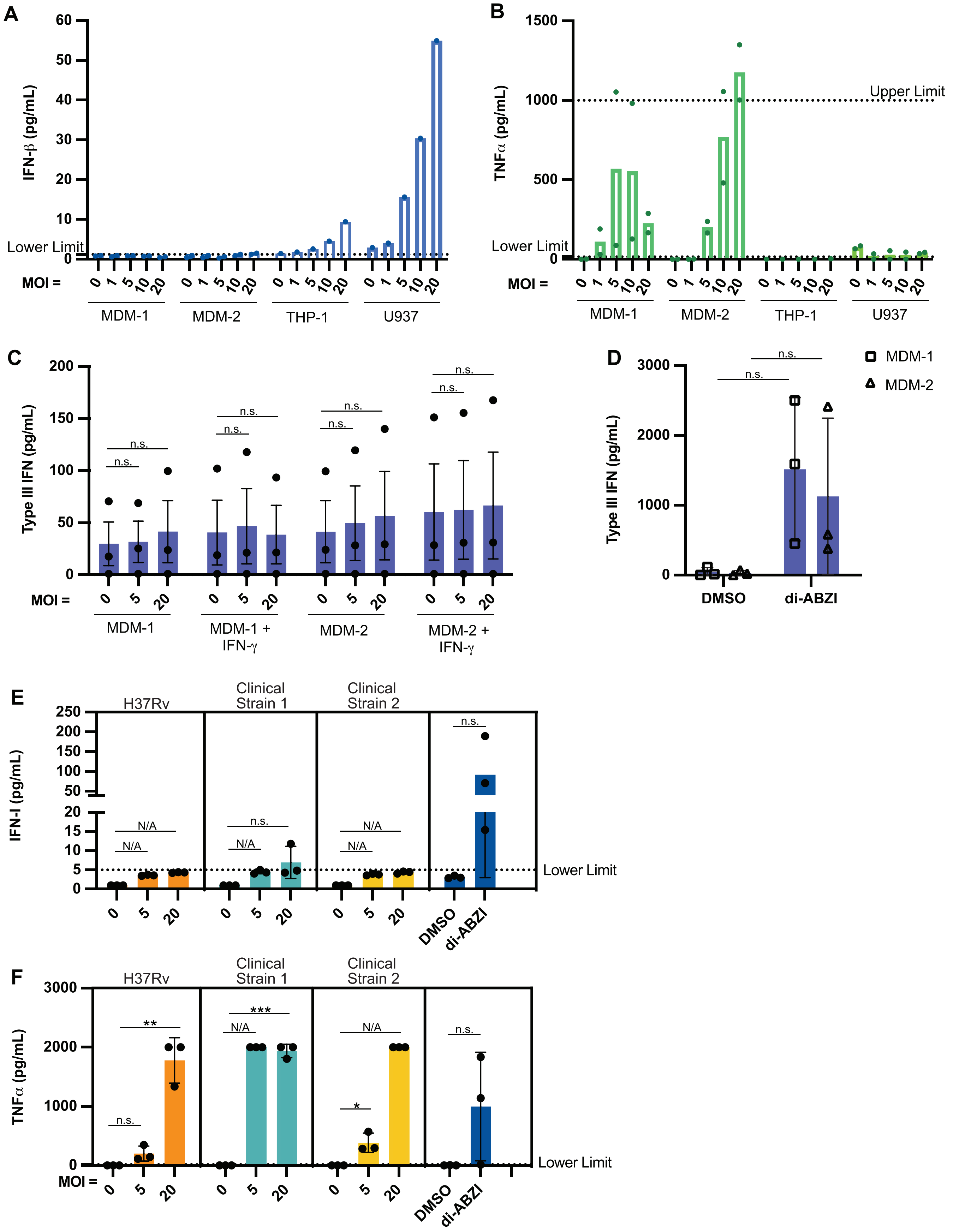
A
IFN-β (pg/mL)
60
50
40
30
20
10
Lower Limit
0
MOI =
0 1 5 10 20
MDM-1
MDM-2
THP-1
U937
B
TNFα (pg/mL)
1500
1000
500
Upper Limit
Lower Limit
MDM-1
MDM-2
THP-1
U937
C
Type III IFN (pg/mL)
200
150
100
50
n.s.
MOI =
0 5 20
MDM-1
MDM-1 + IFN-γ
MDM-2
MDM-2 + IFN-γ
D
3000
2000
1000
MDM-1
MDM-2
DMSO
di-ABZI
E
IFN-I (pg/mL)
H37Rv
Clinical Strain 1
Clinical Strain 2
250
200
150
100
50
20
15
10
5
N/A
Lower Limit
F
TNFα (pg/mL)
**
***
*

Figure EV2. Mtb-infected MDMs do not secrete more IFN-III than uninfected MDMs at 1 d post infection nor detectable IFN-I upon infection with clinical isolates of Mtb.

MDM-1 and MDM-2 ($n = 2$) and 10 ng/mL PMA differentiated THP-1 cells and U937 cells were infected with Mtb H37Rv. 1 d post infection, supernatants were collected. (**A**) IFN-β was measured by a high sensitivity ELISA. (**B**) TNFα was measured by ELISA. (**C**, **D**) MDM-1 and MDM-2 ($n = 3$) were primed with or without 2.5 ng/mL IFN-γ for 1 day and then (**C**) infected with Mtb strain H37Rv (**C**) or (**D**) treated with 3 μM di-ABZI. Supernatant was collected 1 d post infection. IFN-III expression in the supernatant was measured via the IFN-III HEKBlue reporter assay and quantified by performing a sigmoidal, 4 parameter interpolation from an IFN-λ1 (IL-29) standard curve. (**E**, **F**) MDM-2 ($n = 3$) were differentiated for only 1 week before infection with Mtb H37Rv, clinical strain 1, clinical Mtb strain 2, or treatment with 3 μM ABZI. (**E**) IFN-I expression in the supernatant was measured via the IFN-I HEKBlue reporter assay, and (**F**) TNFα expression was measured via ELISA. Data Information: (**A**–**F**) Bar graphs report the mean ± SEM. Each • represents an individual donor with 2–3 technical replicates per donor. Values too low to be interpolated were assigned a value of 1 pg/mL. Statistical significance was determined using a one-tailed paired, *t*-test (*$p < 0.5$; **$p < 0.01$; ***$p < 0.001$; n.s. indicates no significance; N/A indicates not applicable). N/A was assigned if all values between two groups being tested were below the limit of detection (**E**) or if it was not possible to compute *t*-test because all values per group were the same (**F**). In each panel, *n* indicates biological replicates.

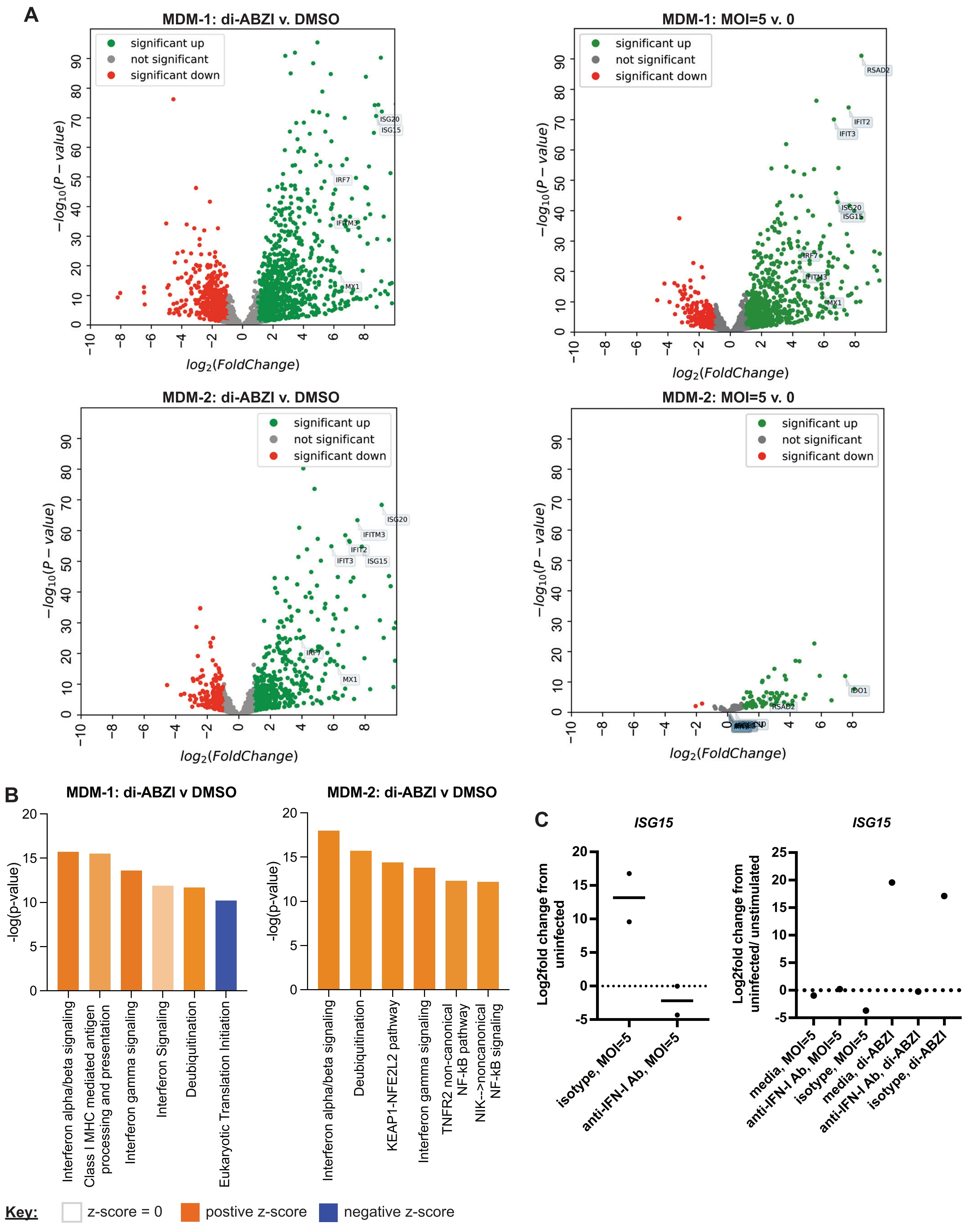
A
MDM-1: di-ABZI v. DMSO
MDM-1: MOI=5 v. 0
MDM-2: di-ABZI v. DMSO
MDM-2: MOI=5 v. 0
significant up
not significant
significant down
−log10(P − value)
log2(FoldChange)
ISG20
ISG15
IRF7
IFITM3
MX1
RSAD2
IFIT2
IFIT3
IDO1
B
MDM-1: di-ABZI v DMSO
MDM-2: di-ABZI v DMSO
-log(p-value)
Interferon alpha/beta signaling
Class I MHC mediated antigen processing and presentation
Interferon gamma signaling
Interferon Signaling
Deubiquitination
Eukaryotic Translation Initiation
KEAP1-NFE2L2 pathway
TNFR2 non-canonical NF-kB pathway
NIK-->noncanonical NF-kB signaling
Key:
z-score = 0
postive z-score
negative z-score
C
ISG15
Log2fold change from uninfected
Log2fold change from uninfected/ unstimulated
isotype, MOI=5
anti-IFN-I Ab, MOI=5
media, MOI=5
media, di-ABZI
anti-IFN-I Ab, di-ABZI
isotype, di-ABZI

 © The Author(s)

◀ **Figure EV3. Mtb-infected MDM-1 upregulate ISGs in an IFN-I dependent manner.**

(**A**) MDM-1 and MDM-2 ($n = 3$) were infected with Mtb H37Rv or treated with 3 μM di-ABZI or vehicle controls (equal volume of DMSO as in 3 μM di-ABZI). Lysates were collected 1 day post-treatment or post-infection for RNA sequencing. Volcano plots show log_2fold changes of ISGs determined by Interferome between stimulated/infected MDM-1 compared to unstimulated MDM-1 and the corresponding $-log_{10}$ adjusted *p*-values. (**B**) Ingenuity Pathway Analysis (QIAGEN) of the top 6 canonical pathways upregulated in di-ABZI treated MDM-1 (left) and di-ABZI treated MDM-2 (right) determined by -log(adjusted *p*-value) and absolute z-scores >3. (**C**) Mtb-infected MDM-1 from 2 donors (left) and 1 donor (right) were treated with anti-IFN-I neutralizing Abs or IgG2a isotype Ab for 2 h pre-infection, infected with Mtb H37Rv and again treated with the respective Ab after Mtb was washed out 4 h post infection. Lysates were collected 1 d post infection. *ISG15* expression was measured by RT-qPCR. Data Information: (**A**) Each • represents 1 gene. Statistical significance was determined using the DESeq2 package, which uses the Wald test and Benjamini–Hochberg method to correct the FDR (**A**), and using QIAGEN's IPA analysis, which employs Fisher's exact test and the Benjamini–Hochberg method to control the false discovery rate (Krämer et al, 2013) (**B**). (**C**) Each • represents the average value of 4 technical replicates per donor, while *n* indicates biological replicates.

© The Author(s)

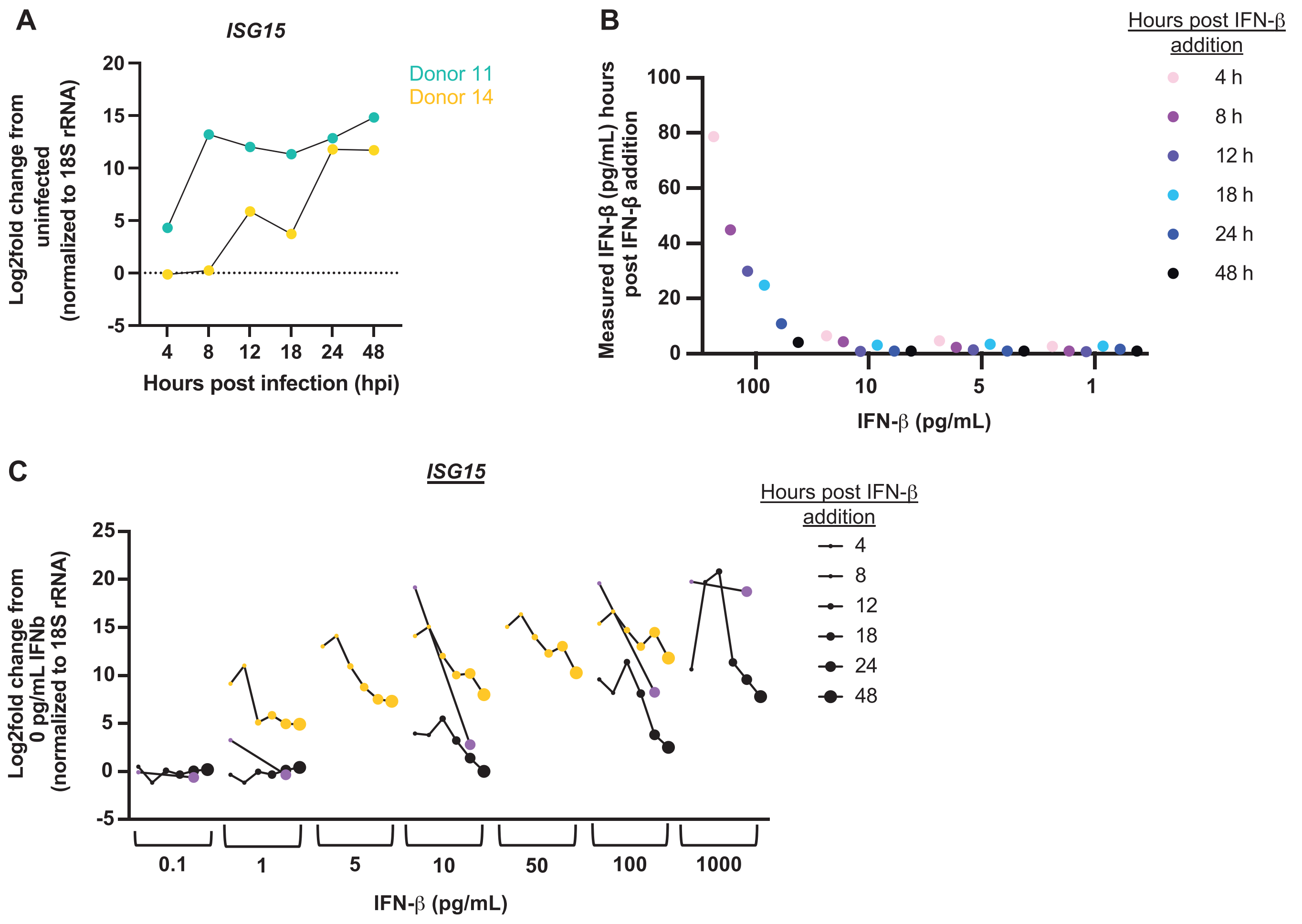

Figure EV4. MDM-1 consume IFN-β.

(**A**) MDM-1 ($n = 2$) were infected with Mtb H37Rv and lysates were collected at indicated timepoints. *ISG15* expression was measured by RT-qPCR. (**B**) MDM-1 ($n = 1$) were stimulated with 1–100 pg/mL IFN-β, supernatants were collected at indicated timepoints, and IFN-I expression was measured via the IFN-I HEKBlue reporter assay. IFN-I was quantified by performing a sigmoidal, 4 parameter interpolation from an IFN-β standard curve. (**C**) MDM-1 ($n = 1$–3) were treated with IFN-β and lysates were collected at indicated timepoints. *ISG15* expression was measured by RT-qPCR. Each • represents the average value of 3–4 technical replicates per donor, while *n* indicates biological replicates. Data Information: Each • represents the average value of 3–4 technical replicates per donor (**A**), the average value of 2 technical replicates per donor (**B**), and the average value of 3–4 technical replicates per donor (**C**), *n* indicates biological replicates.

 © The Author(s)

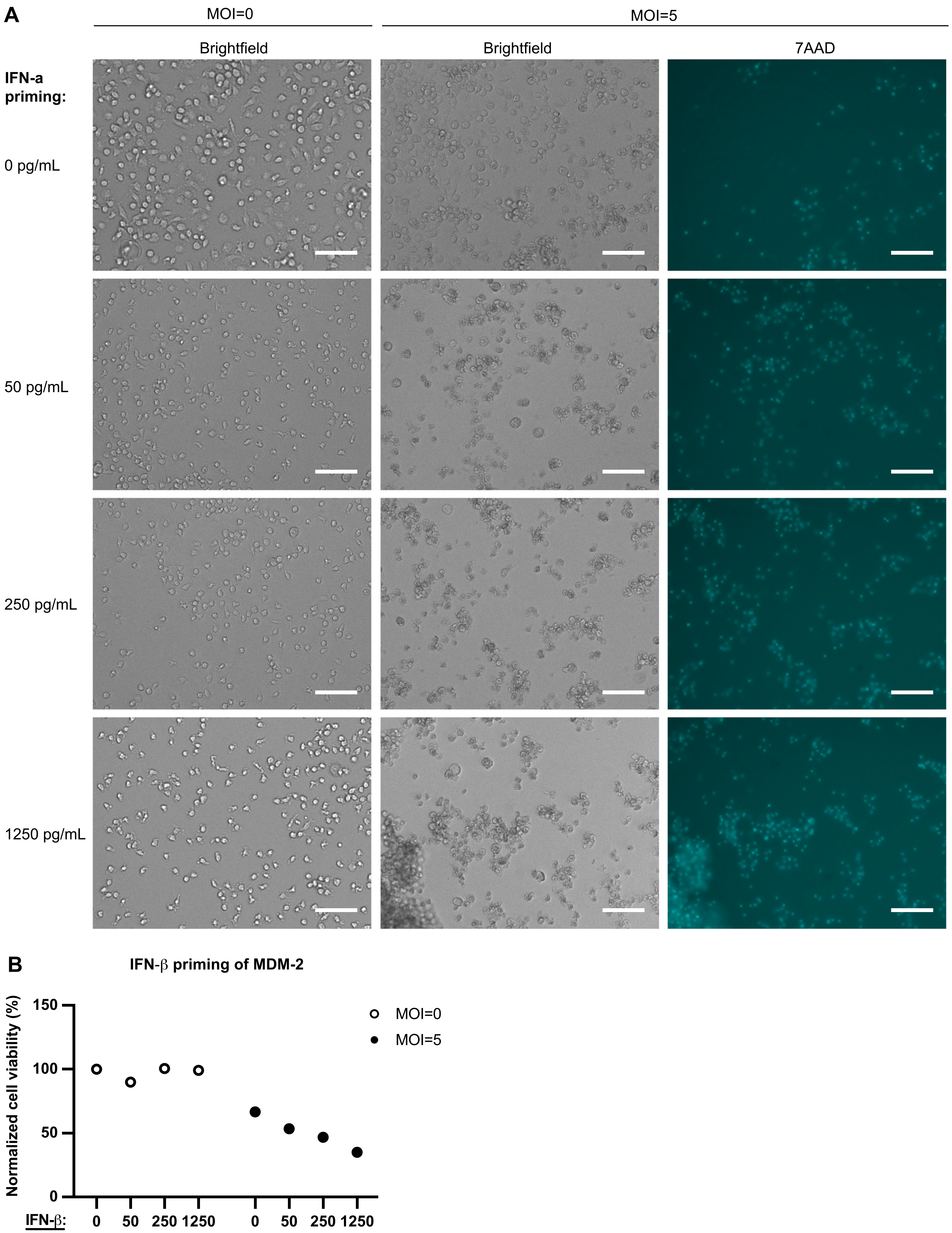
A
MOI=0
MOI=5
Brightfield
Brightfield
7AAD
IFN-a priming:
0 pg/mL
50 pg/mL
250 pg/mL
1250 pg/mL
B
IFN-β priming of MDM-2
Normalized cell viability (%)
150
100
50
0
MOI=0
MOI=5
IFN-β:
0
50
250
1250
0
50
250
1250

Figure EV5. Exogenous IFN-I exacerbates Mtb-induced MDM death.

(**A**) Representative images of MDM-1 ($n = 1$) that were primed with IFN-α 1 day pre-infection, infected with Mtb H37Rv, stained with 7-aminoactinomycin D (7-AAD) and imaged at 10×2 d post infection. Scale bars = 100 μm. (**B**) MDM-2 ($n = 1$) were primed with IFN-β 1 day pre-infection, infected with Mtb H37Rv, and cell viability was measured via CellTiter Glo assay. Data Information: (**B**) Dot plot reports mean value of 3 technical replicates per 1 donor, *n* indicates biological replicates.

 © The Author(s)
